# Mechanisms of Regulation of Olfactory Transduction and Adaptation in the Olfactory Cilium

**DOI:** 10.1371/journal.pone.0105531

**Published:** 2014-08-21

**Authors:** Gabriela Antunes, Ana Maria Sebastião, Fabio Marques Simoes de Souza

**Affiliations:** 1 Institute of Pharmacology and Neurosciences, Faculty of Medicine, University of Lisbon, Lisbon, Portugal; 2 Neurosciences Unit, Institute of Molecular Medicine, University of Lisbon, Lisbon, Portugal; 3 Center for Mathematics, Computation and Cognition, Federal University of ABC, São Bernardo do Campo, Brazil; 4 Laboratory of Neural Systems, Psychobiology Sector, Department of Psychology, Faculdade de Filosofia, Ciências e Letras de Ribeirão Preto, Universidade de São Paulo, Ribeirão Preto, Brazil; Tokai University, Japan

## Abstract

Olfactory adaptation is a fundamental process for the functioning of the olfactory system, but the underlying mechanisms regulating its occurrence in intact olfactory sensory neurons (OSNs) are not fully understood. In this work, we have combined stochastic computational modeling and a systematic pharmacological study of different signaling pathways to investigate their impact during short-term adaptation (STA). We used odorant stimulation and electroolfactogram (EOG) recordings of the olfactory epithelium treated with pharmacological blockers to study the molecular mechanisms regulating the occurrence of adaptation in OSNs. EOG responses to paired-pulses of odorants showed that inhibition of phosphodiesterases (PDEs) and phosphatases enhanced the levels of STA in the olfactory epithelium, and this effect was mimicked by blocking vesicle exocytosis and reduced by blocking cyclic adenosine monophosphate (cAMP)-dependent protein kinase (PKA) and vesicle endocytosis. These results suggest that G-coupled receptors (GPCRs) cycling is involved with the occurrence of STA. To gain insights on the dynamical aspects of this process, we developed a stochastic computational model. The model consists of the olfactory transduction currents mediated by the cyclic nucleotide gated (CNG) channels and calcium ion (Ca^2+^)-activated chloride (CAC) channels, and the dynamics of their respective ligands, cAMP and Ca^2+^, and it simulates the EOG results obtained under different experimental conditions through changes in the amplitude and duration of cAMP and Ca^2+^ response, two second messengers implicated with STA occurrence. The model reproduced the experimental data for each pharmacological treatment and provided a mechanistic explanation for the action of GPCR cycling in the levels of second messengers modulating the levels of STA. All together, these experimental and theoretical results indicate the existence of a mechanism of regulation of STA by signaling pathways that control GPCR cycling and tune the levels of second messengers in OSNs, and not only by CNG channel desensitization as previously thought.

## Introduction

As we expand our knowledge on cell signaling, it becomes increasingly clear that the complexity of the cellular systems cannot be fully understood through the investigation of isolated molecules, but as a function that emerges from a molecular network temporally and spatially highly orchestrated [1], [2]. Therefore, the comprehension of cellular systems requires a systems biology approach, which includes not only the identification of their components, but also the study of the systems structures and dynamics, and the mechanisms that control and modify their properties [3]. The systems-level approach has been used successfully in the investigation of several different processes in which the complexity of the molecular interactions challenges their understanding through classical reductionist approaches [4]. Among these processes is the olfactory transduction and adaptation, two key cellular events involved in odorant reception and in its consequences for finding food, avoiding predators, identifying sexual partners and other relevant aspects of animal survival [5]–[7].

The olfactory information is transmitted through the signaling pathways located in the cilia of olfactory sensory neurons (OSNs). Odorants bind to G protein coupled receptors (GPCRs) triggering the activation of G_αolf_ that in turn activates adenylate cyclase 3 (AC3) producing cyclic adenosine monophosphate (cAMP) [8]. Molecules of cAMP bind to cyclic nucleotide-gated (CNG) channels promoting their opening and allowing cations, mainly sodium (Na^+^) and calcium (Ca^2+^) ions, to flow to the intracellular medium depolarizing the cell. This transient increase of the intracellular Ca^2+^ concentration ([Ca^2+^]) opens Ca^2+^-activated chloride (CAC) channels that amplify the CNG channel signal [9].

The OSN adapts after a previous exposure to stimulus. Short-term adaptation (STA) is defined as a decrease in responsiveness to the odor presentation that occurs within a few seconds after a brief conditioning stimulus. Previous works have shown that STA is induced by a brief odor pulse, has a recovery time of seconds, and can be abolished by the removal of intracellular Ca^2+^ [10]. Moreover, experimental and theoretical evidences have demonstrated that STA happens at the level of CNG channel [11] and presumably is independent of the activity of phosphodiesterases (PDEs), which hydrolyze cAMP terminating its action [12]. However, Ca^2+^-dependent mechanisms involving PDEs may act by modulating the concentration of cAMP with consequences for STA [13], [14]. Additionally, Ca^2+^/Calmodulin (Ca^2+^/CaM) can bind to CNG channels, promoting a decrease in its affinity to cAMP [15], a process that seems to be involved in STA. Since cAMP opens CNG channels leading to Ca^2+^ influx that induces STA [11], Ca^2+^ extrusion mainly through Na^+^/Ca^2+^ and K^+^ exchangers (NCKX) [16]–[18] and, possibly, plasma membrane Ca^2+^-ATPases (PMCA) [19]–[22] leads to the resetting the cellular resting state. There are evidences that K^+^-independent Na^+^/Ca^2+^ exchangers (NCX) and NCKX are present in mammalian OSNs [23], [24].

Another mechanism controlling intracellular concentrations of cAMP is the internalization of GPCRs mediated by phosphorylation catalyzed by cAMP-dependent protein kinase (PKA) [25] and G-protein-coupled receptor kinase 3 (GRK3) [26], [27], but the specific role of GPCR cycling in the occurrence of STA is unknown. In addition, although cAMP and Ca^2+^ are involved in STA, it is uncertain how their regulation by distinct signaling pathways affects the recovery times of OSN responses to odorants.

The decrease in OSN sensitivity can occur not only in the presence of a brief conditioning stimulus, but also in the presence of a prolonged stimulus. The decline in OSN response produced by a sustained odor pulse is defined as desensitization (DS). The molecular mechanism behind the occurrence of DS likely act upstream of cAMP production and involves Ca^2+^ dependent pathways [28].

The present study uses pharmacological manipulation of signaling pathways involved in STA accessed by electro-olfactogram (EOG) responses to paired pulses of odorant to investigate the action of PDE, PKA, and vesicle internalization and recycling in the fine control of olfactory adaptation. In addition, we have developed a stochastic kinetic model of STA to gain insights on its dynamical aspects. Previous attempts for modeling olfactory transduction and adaptation in OSNs have generated important conceptual advances in the field [29]–[37]. However, the overwhelming accumulation of experimental data constantly challenging our comprehension of this system urges for a unifying approach to achieve a better understanding of olfactory transduction and adaptation. In this way, we have developed a stochastic kinetic model strongly constrained by the available experimental parameters that incorporates detailed kinetic schemes of binding, gating, desensitization and inactivation of CNG and CAC channels along with the dynamics of their ligands, cAMP and Ca^2+^. The model simulates the occurrence of STA in the noise environment of a single olfactory cilium [38], [39] and demonstrates how it promotes the EOG signal. Thus, the model was used for the interpretation of measured drug effects on the odorant responses in the intact olfactory epithelium. The results obtained with the stochastic kinetic model and the pharmacological experiments indicate that STA is not only controlled by desensitization of CNG channels through Ca^2+^/CaM, but also by desensitization and resensitization of GPCRs regulated through signaling pathways that are involved in vesicle cycling.

## Results

**Figure 1:**
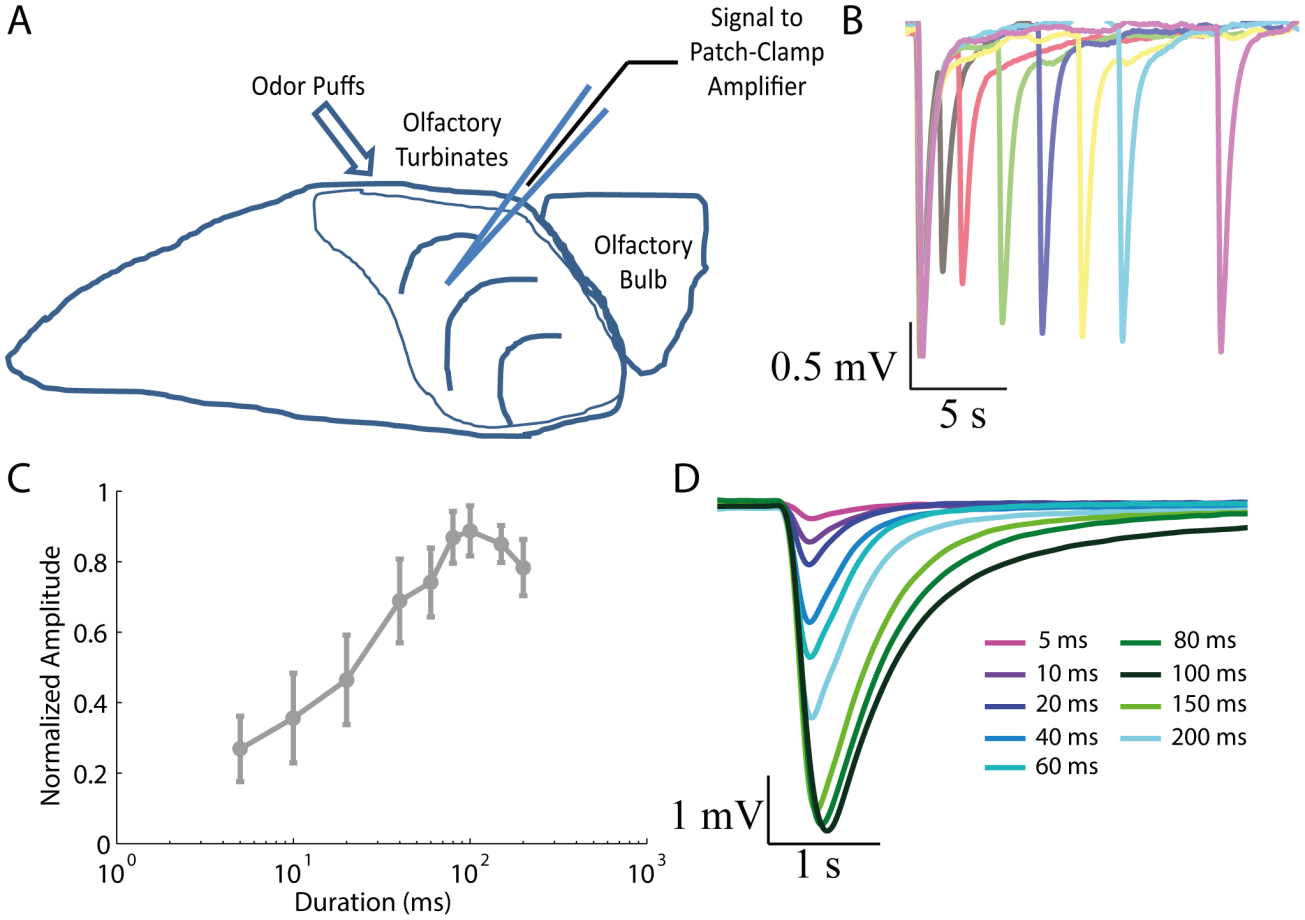
Experimental preparation developed to study the regulation of STA in the intact olfactory epithelium. A: The odorants are puffed in the surface of the olfactory epithelium to observe resulting potential changes caused by individual responsible OSNs cilia in the recording field. B: Representative EOG responses to 100 ms paired-pulses of odorant stimulation. There is a gradual recovery from adaptation along with the increase of the ISI (1, 2, 4, 6, 8, 10, and 15 s). C: Dose-response relation of EOG responses averaged from five rats to odorant puffs of increasing duration. D: Representative EOG responses to odorant puffs of increasing duration.

Olfactory adaptation and desensitization are generally classified by distinct onset and recovery time, and by their differential pharmacological properties. In this work, we have investigated EOG responses (Figure 1A) in the presence of pharmacological inhibitors of key enzymes that are located in the olfactory sensory cilia, which is the cellular compartment that encloses the signaling pathways involved in olfactory adaptation. We used brief odorant stimulation to activate mostly the signaling pathways involved with STA. The role of specific components of the signaling pathways was investigated by comparing the effects of the pharmacological treatments in the EOG parameters and STA, which was defined as a decline in responsiveness to the odor presentation that occurs within a few seconds after a short conditioning stimulus of 100 ms and has a recovery time faster than 30 s [40]. A typical feature of STA is a shift of the stimulus-response relation when the curve is fitted with the Hill equation. Thus, we measured the effects of each drug on STA by fitting curves of Inter-Stimuli Interval (ISI) versus the percent of recovery of adaptation (PRA) using the Hill equation [41]:
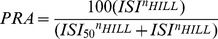
(1)


where *ISI_50_* and *n_HILL_* stand for the half-maximum ISI and the Hill coefficient, respectively. These two parameters were statistically compared across the pharmacological treatments. Additionally, we tested the effects of the pharmacological treatments on the kinetic parameters of EOG responses to odorants, namely the response latency (L), rise time (RT), and decay time (DT). Representative EOG responses to paired pulses of odorants in control condition are shown in Figure 1B. There is a typical decline in the response to the second odorant pulse relative to the first and a recovery from the reduced responsiveness by increasing the ISI between the first and second odorant stimulus. The intensity of EOG responses increased with the duration of odorant puffs and declined slightly in the peak at very long durations (Figure 1C). A representative EOG response to odorant puffs of increasing duration is shown in Figure 1D. We observed that DS is particularly involved in the reduction of EOG responses to paired odorant puffs of increasing duration (Figure S1 in File S1), which was confirmed by a significant reduction with p<0.05 in the maximum EOG responses to the second stimulus (0.53 [0.48, 0.57]) in comparison to the EOG responses to the first stimulus (1.01 [0.85, 1.16]), where the brackets indicate the confidence interval of 95% used to fit the data with the Hill equation. We minimized the occurrence of DS by using only brief odorant puffs with 100 ms of duration to investigate the role of key enzymes involved in STA.

The drugs tested were N-[2-(p-Bromocinnamylamino)ethyl]-5-isoquinolinesulfonamide (H89), dynasore, 3-isobutyl-1-methylxanthine (IBMX), okadaic acid, and monensin that disrupt selectively the enzymatic activity of PKA, dynamin GTPase, PDEs, phosphatases, and GPCR recycling, respectively. Statistical analysis, using one-way ANOVA test, revealed that *n* did not differ but *ISI_50_* differed significantly across all treatments, F(7,44) = 1.7368, p = 0.1251, and F(7,44) = 7.9476, p<0.0001, respectively. In addition the latency, rise time and decay time differed significantly across all treatments F(28, 1248.9) = 24.384, p<0.0001. All together, these results suggest that the treatments affected the recovery time from STA and the kinetics of EOG responses.

### PDE inhibition affects STA and this effect is mimicked by phosphatase inhibition

**Figure 2:**
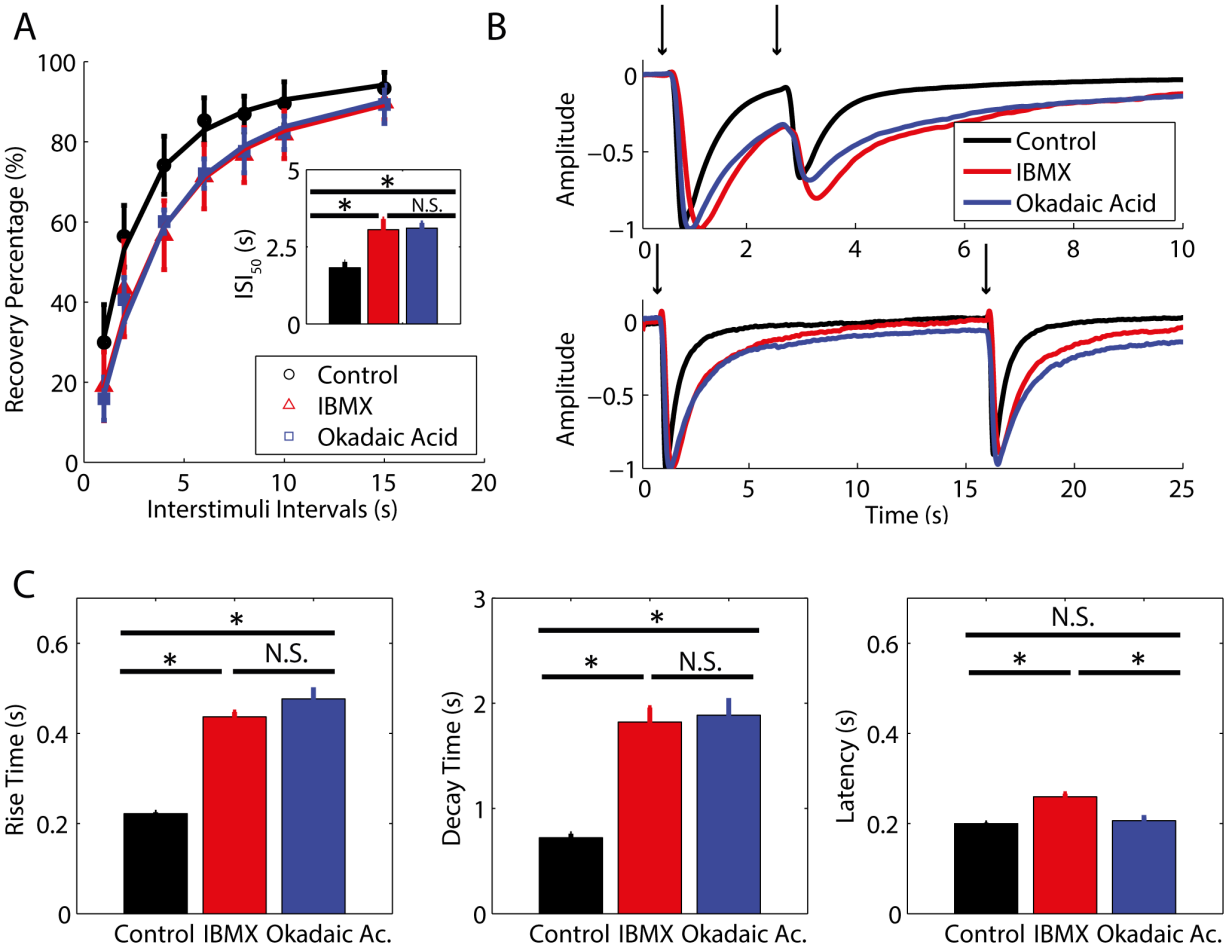
PDE inhibition affects STA and this effect is mimicked by phosphatase inhibition. A: The influence of IBMX and okadaic acid on STA was verified by the averaged percent of recovery from adaptation for each experimental condition. The intervals between 100 ms paired pulses of odorants are plotted versus the percent of recovery from adaptation. The parameters for fitting the curves are n_Hill_control_ = 1.33, and ISI_50_control_ = 1.82 s, n_Hill_IBMX_ = 1.33, and ISI_50_IBMX_ = 3.05 s, n_Hill_okadaic_ = 1.40, and ISI_50_okadaic_ = 3.10 s. The inset shows the ISI for half-maximum recovery. B: Representative traces of EOG responses during the occurrence of adaptation for an ISI of 2 s (top panel) and during full recovery of adaptation for an ISI of 15 s (bottom panel). The voltage of EOG responses was normalized for comparison. Arrows indicate the timing of the stimulus. C: Parameters of EOG responses to a single 100 ms pulse of odorant. The left panel (rise time), middle panel (decay time) and right panel (latency) show the averaged parameters for each pharmacological treatment. Statistically significant differences (*) at P<0.05 and non-significant differences (N.S.) are indicated.

Tukey's HSD *post-ho*c comparisons indicate that the IBMX-treated group (Mean *ISI_50_* (M_ISI50_) = 3.05 s, 95% Confidence interval (CI) [2.48 s, 3.62 s]) and okadaic acid-treated group (M_ISI50_ = 3.10 s, 95% CI [2.57 s, 3.63 s]) had a significantly higher *ISI_50_* than the control group (M_ISI50_ = 1.82 s, 95% CI [1.29 s, 2.35 s]), p = 0.0083 and p = 0.003, respectively (Figure 2A). These results indicate that the blockage of PDEs and phosphatases strengthens STA occurrence. The difference between the *ISI_50_* of the IBMX-treated and the okadaic acid-treated groups were not statistically significant at p<0.05. In addition, IBMX-treated group (M_RT_ = 0.4364 s, 95% CI [0.40 s, 0.46 s] and M_DT_ = 1.82 s, 95% CI [1.63 s, 2.00 s]) and okadaic acid-treated group (M_RT_ = 0.47 s, 95% CI [0.45 s, 0.50 s], and M_DT_ = 1.88 s, 95% CI [1.71 s, 2.06 s]) had a significant higher rise time and decay time in comparison to the control group (M_RT_ = 0.22 s, 95% CI [0.19 s, 0.24 s], p<0.0001 for both comparisons and M_DT_ = 0.72 s, 95% CI [0.54 s, 0.89 s], p<0.0001 for both comparisons) (Figure 2B and 2C). Thus, the blockage of PDEs and phosphatases slows down the kinetics of EOG responses. The rise time and decay time of the IBMX-treated group compared to the okadaic acid-treated group were not statistically significant at p<0.05, and the latency of the IBMX-treated group (M_L_ = 0.25 s, 95% CI [0.24 s, 0.27 s]) were significantly higher than the control group (M_L_ = 0.19 s, 95% CI [0.18 s, 0.21 s]) and okadaic acid-treated group (M_L_ = 0.20 s, 95% CI [0.19 s, 0.21 s], p<0.0001 for both comparisons (Figure 2B and 2C). The latency of the okadaic acid-treated group (M_L_ = 0.20 s, 95% CI [0.19 s, 0.21 s]) was not statistically different from the control group (M_L_ = 0.19 s, 95% CI [0.18 s, 0.21 s]) at p<0.05.

Okadaic acid mimicked all effects of IBMX excluding the latency. The similar effects of IBMX and okadaic acid suggest that targets acting downstream from the cAMP production are involved in the regulation of STA. As IBMX increases cAMP intracellular level by blocking PDE1C and PDE4A [14], it is likely that the PKA activation might be involved with STA occurrence. Therefore, IBMX and okadaic acid might present similar results because the blockage of PDEs contributes to PKA activation and, consequently, the phosphorylation of its targets. In a similar manner, the inhibition of the phosphatases, which are the enzymes that counteract the action of protein kinases, would also increase the phosphorylation of PKA targets.

### PKA and dynamin inhibition partially prevented the outcome of PDE inhibition

**Figure 3:**
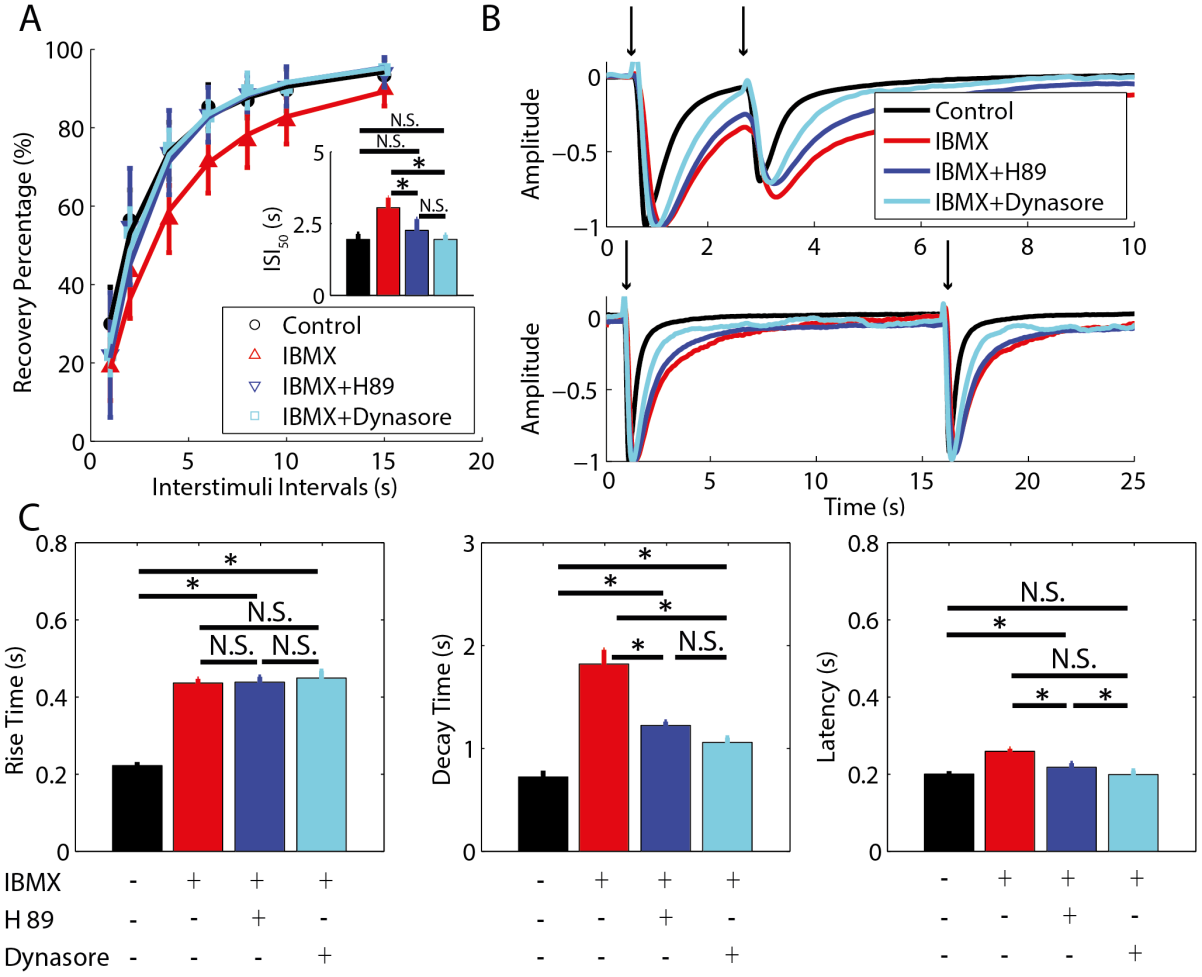
PKA and dynamin inhibition partially prevented the outcome of PDE inhibition. A: The effect of IBMX, IBMX+H89 and IBMX+dynasore on STA was verified by the averaged percent of recovery from adaptation for each experimental condition. The intervals between 100 ms paired pulses of odorants are plotted versus the percent of recovery from adaptation. The parameters for fitting the curves are n_Hill_IBMX_ = 1.33, and ISI_50_IBMX_ = 3.05 s, n_Hill_IBMX+H89_ = 1.60, and ISI_50_IBMX+H89_ = 2.26 s, n_Hill_IBMX+dynasore_ = 1.52, and ISI_50_IBMX+dynasore_ = 1.94 s. The inset shows the ISI for half-maximum recovery. B: Representative traces of EOG responses during the occurrence of adaptation for an ISI of 2 s (top panel) and during full recovery of adaptation for an ISI of 15 s (bottom panel). The voltage of EOG responses was normalized for comparison. Arrows indicate the timing of the stimulus. C: Parameters of EOG responses to a single 100 ms pulse of odorant. The left panel (rise time), middle panel (decay time) and right panel (latency) show the averaged parameters for each pharmacological treatment. Statistically significant differences (*) at P<0.05 and non-significant differences (N.S.) are indicated.

To verify the putative targets of PDE inhibition, we used a cocktail of IBMX with H89 to inhibit PKA and PDE simultaneously. In addition, we tested a combination of IBMX with dynasore to disrupt the effects of PKA phosphorylation on GPCR internalization simultaneously to the inhibition of PDE [26], [27]. Fisher's LSD *post-hoc* analyses reveal that the IBMX+dynasore-treated group (M_ISI50_ = 1.94 s, 95% IC [1.32 s, 2.57 s]) and IBMX+H89-treated group (M_ISI50_ = 2.26 s, 95% IC [1.69 s, 2.83 s]) had a significant reduction of the *ISI_50_* in comparison to the IBMX-treated group (M_ISI50_ = 3.05 s, 95% CI [2.48 s, 3.62 s]), p = 0.0026 and p = 0.0214, respectively. The *ISI_50_* of the IBMX+H89-treated group (M_ISI50_ = 2.26 s, 95% IC [1.69 s, 2.83 s]) did not differ from the IBMX+dynasore-treated group (M_ISI50_ = 1.94 s, 95% IC [1.32 s, 2.57 s]), p = 0.3682 (Figure 3A). In addition, *ISI_50_* of IBMX+dynasore-treated group (M_ISI50_ = 1.94 s, 95% IC [1.32 s, 2.57 s]) and IBMX+H89-treated group (M_ISI50_ = 2.26 s, 95% IC [1.69 s, 2.83 s]) did not differ from control group (M_ISI50_ = 1.82 s, 95% IC [1.35 s, 2.29 s]), p = 0.7131 and p = 0.1748, respectively. Taking together, these results indicate that PKA inhibition prevents the effects obtained with the blockage of PDEs. Moreover, the results obtained with dynasore suggests that PKA is acting on GPCR internalization during STA. In consequence, blocking GPCR internalization prevents the effect of PDEs inhibition. Both the IBMX+dynasore-treated group (M_L_ = 0.19 s, 95% CI [0.18 s, 0.21 s]) and the IBMX+H89-treated group (M_L_ = 0.21 s, 95% CI [0.20 s, 0.23 s]) significantly reduced the increase in the latency of EOG responses caused by IBMX (M_L_ = 0.25 s, 95% CI [0.24 s, 0.27 s]), p<0.0001 for both comparisons (Figure 3B and 3C); and did not differed significantly from the control group (M_L_ = 0.19 s, 95% CI [0.18 s, 0.21 s]), p = 0.9570 and p = 0.0545, respectively. The latency of the IBMX+dynasore-treated group was not significantly different from the IBMX+H89-treated group, p = 0.0872. In addition, IBMX+dynasore-treated group (M_DT_ = 1.06 s, 95% CI [0.83 s, 1.28 s]) and the IBMX+H89-treated group (M_DT_ = 1.22 s, 95% CI [1.03 s, 1.41 s]) significantly diminished the decay time of EOG responses observed with IBMX (M_DT_ = 1.82 s, 95% CI [1.63 s, 2.00 s]), p<0.0001 for both comparisons (Figure 2B and 2C); but they were significantly different from control (M_DT_ = 0.72 s, 95% CI [0.55 s, 0.88 s]), p = 0.0170 and p<0.0001, respectively. The latency of IBMX+dynasore-treated group was not significantly different from the IBMX+H89-treated group, p = 0.2633. Neither the IBMX+dynasore-treated group (M_RT_ = 0.44 s, 95% CI [0.41 s, 0.46 s]) and the IBMX+H89-treated group (M_RT_ = 0.43 s, 95% CI [0.41 s, 0.46 s]) have affected significantly at p<0.05 the rise time increased by IBMX (M_RT_ = 0.43 s, 95% CI [0.40 s, 0.46 s]). The rise time of the IBMX+dynasore-treated group and IBMX+H89-treated group differed significantly from the control group (M_RT_ = 0.22 s, 95% CI [0.21 s, 0.22 s]), p<0.0001 for both comparisons, but did not differ significantly from each other at p<0.05. The decrease in the latency and decay time observed in the groups treated with IBMX+H89 and IBMX+dynasore in comparison to the results observed in the group treated with IBMX indicates that blocking PKA or dynasore simultaneously with the PDE inhibition reduce the IBMX effect.

**Figure 4:**
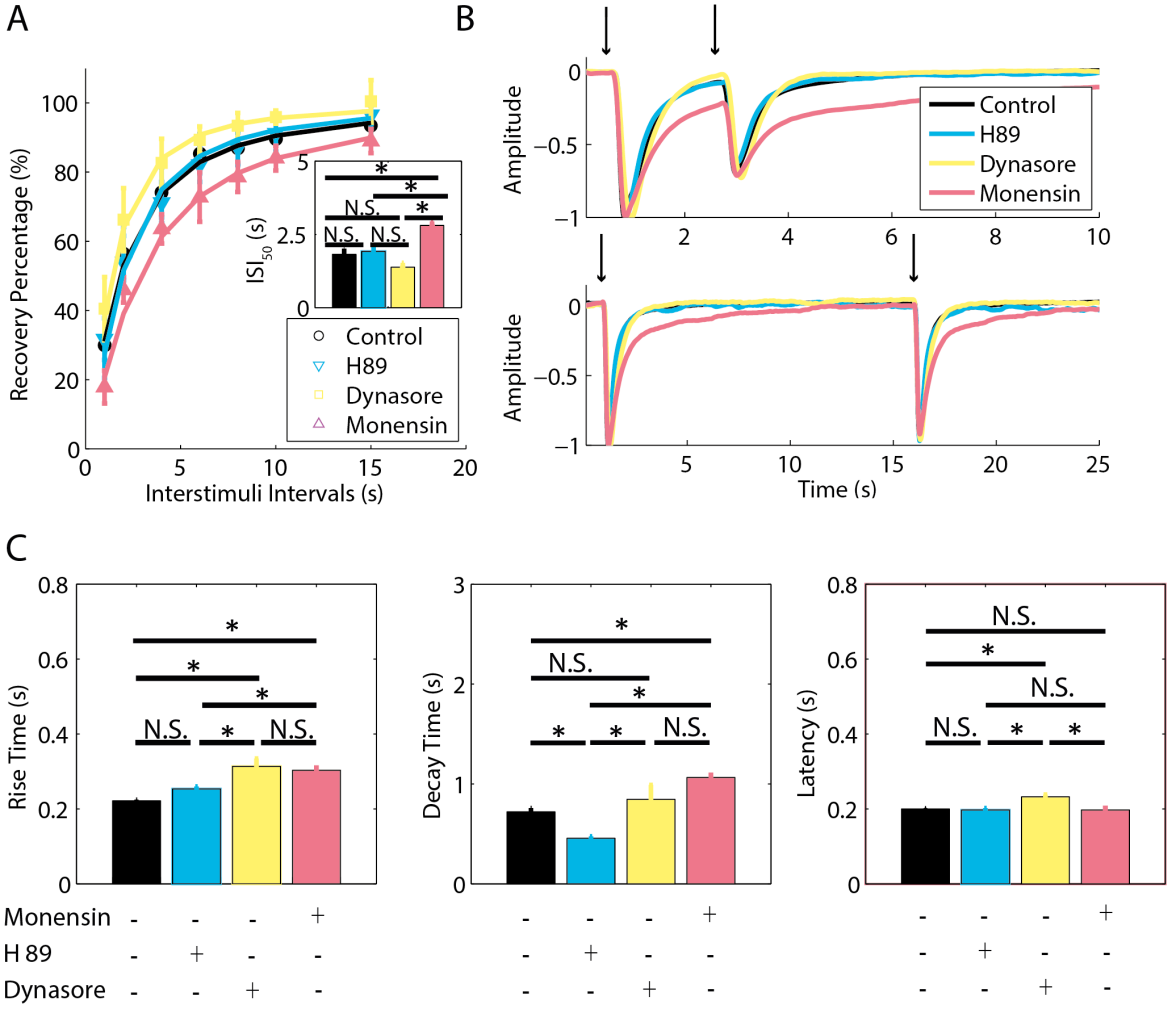
Inhibition of PKA, dynamin, and vesicle exocytosis. A: The effect of H89, dynasore and monensin on STA was assessed by the averaged percent of recovery from adaptation for each experimental condition. The intervals between 100 ms paired pulses of odorants are plotted versus the percent of recovery from adaptation. The parameters for fitting the curves are n_Hill_control_ = 1.33, and ISI_50_control_ = 1.82 s, n_Hill_H89_ = 1.50, and ISI_50_H89_ = 1.92 s, n_Hill_dynasore_ = 1.56, and ISI_50_dynasore_ = 1.38 s, n_Hill_monensin_ = 1.31, and ISI_50_monensin_ = 2.80 s. The inset shows the ISI for half-maximum recovery. B: Representative traces of EOG responses during the occurrence of adaptation for an ISI of 2 s (top panel) and during full recovery of adaptation for an ISI of 15 s (bottom panel). The voltage of EOG responses was normalized for comparison. Arrows indicate the timing of the stimulus. C: Parameters of EOG responses to a single 100 ms pulse of odorant. The left panel (rise time), middle panel (decay time) and right panel (latency) show the averaged parameters for each pharmacological treatment. Statistically significant differences (*) at P<0.05 and non-significant differences (N.S.) are indicated.

To verify whether PKA action on STA is independent of PDEs, we treated the olfactory epithelium with H89 and dynasore in the absence of IBMX to uncover their isolated effects on EOG. Fisher's LSD *post-ho*c comparisons between the control group and H89 and dynasore-treated groups indicate that these treatments did not affect significantly the *ISI_50_* and did not differ from each other at p<0.05 (Figure 4A), but the dynasore treated group had a small tendency of reducing the *ISI_50_*. Fisher's LSD *post-hoc* analyses revealed that H89-treated group in the absence of IBMX did not affect significantly any parameter of EOG except the decay time at p<0.05 in comparison to control, which indicates that higher cAMP levels are required to activate PKA (Figure 4B and 4C). Thus, the PKA basal activity is not sufficient to regulate STA. On the other hand, dynasore-treated group in the absence of IBMX increased the latency (M_L_ = 0.23 s, 95% CI [0.21 s, 0.24 s]) and rise time (M_RT_ = 0.31 s, 95% CI [0.28 s, 0.34 s]) in comparison to control (M_L_ = 0.19 s,95% CI [0.18 s, 0.21 s] and M_RT_ = 0.22 s, 95% CI [0.19 s, 0.24 s]), p = 0.0006 and p<0.0001, respectively, but without affecting the decay time at p<0.05 (Figure 4B and 4C). Moreover, the dynasore-treated group had a longer latency (M_L_ = 0.23 s, 95% CI [0.21 s, 0.24 s, rise time (M_RT_ = 0.31 s, 95% CI [0.2 , 0.34 s]), and decay time (M_DT_ = 0.84 s, 95% CI [0.66 s, 1.02 s]) in comparison to the H89-treated group (M_L_ = 0.19 s, 95% CI [0.18 s, 0.21 s]; M_RT_ = 0.25 s, 95% CI [0.22 s, 0.28 s]; M_DT_ = 0.45 s, 95% CI [0.27 s, 0.63 s]), p = 0.0004, p = 0.0021, and p = 0.0030, respectively (Figure 4B and 4C).

In addition to dynasore, which disrupts vesicle endocytosis, we also tested the effect of monensin to disrupt vesicle exocytosis. Fisher's LSD *post-ho*c comparisons revealed that monensin-treated-group has a higher *ISI_50_* (M_ISI50_ = 2.80 s, 95% CI [2.42 s, 3.19 s]) in comparison to the control (M_ISI50_ = 1.82 s, 95% IC [1.35 s, 2.29 s]), H89-treated-group (M_ISI50_ = 1.92 s, 95% IC [1.44 s, 2.39 s]), and dynasore-treated group (M_ISI50_ = 1.38 s, 95% IC [0.91 s, 1.85 s]), p = 0.0014, p = 0.0054, p<0.0001, respectively (Figure 4A); and did not differ from IBMX-treated-group (M_ISI50_ = 3.05 s, 95% CI [2.58 s, 3.52 s]) and okadaic acid-treated-group (M_ISI50_ = 3.10 s, 95% CI [2.58 s, 3.52 s]) at p<0.05 (Figure 2A). In addition, the monensin-treated-group increased the rise time (M_RT_ = 0.30 s, 95% CI [0.28 s, 0.32 s]) and decay time (M_DT_ = 1.06 s, 95% CI [0.91 s, 1.21 s]) in comparison to control (M_RT_ = 0.22 s, 95% CI [0.19 s, 0.24 s] and M_DT_ = 0.72 s, 95% CI [0.55 s, 0.88 s]), p<0.0001 and p = 0.0026, respectively, but without affecting the latency (M_L_ = 0.19 s, 95% CI [0.18 s, 0.20 s]) in comparison to control (M_L_ = 0.19 s, 95% CI [0.18 s, 0.21 s]) at p<0.05 (Figure 4C). Moreover, the rise time of the monensin-treated-group (M_RT_ = 0.30 s, 95% CI [0.28 s, 0.32 s]) differed from the H89-treated-group (M_RT_ = 0.25 s, 95% CI [0.22 s, 0.28 s]) with p = 0.005, but did not differ from the dynasore-treated-group (M_RT_ = 0.31 s, 95% CI [0.28 s, 0.34 s]) at p<0.05. The decay time of the monensin-treated-group (M_DT_ = 1.06 s, 95% CI [0.91 s, 1.21 s]) differed from the H89-treated-group (M_DT_ = 0.45 s, 95% CI [0.27 s, 0.63 s]) with p<0.0001, but it did not differ significantly from the dynasore-treated-group (M_DT_ = 0.84 s, 95% CI [0.66 s, 1.02 s]) at p<0.05; and its latency (M_L_ = 0.19 s, 95% CI [0.18 s, 0.20 s]) differed from the dynasore-treated-group (M_L_ = 0.23 s, 95% CI [0.21 s, 0.24 s]) with p = 0.0001, but did not differ from the H89-treated-group (M_L_ = 0.19 s, 95% CI [0.18 s, 0.21 s]) at p<0.05 (Figure 4C).

Considering that okadaic acid and monensin affect vesicle exocytosis [42]–[45] and dynasore affects vesicle endocytosis [46], and GPCR internalization impacts cAMP kinetics in olfactory sensory cilium [26], [27] by down regulating the activation of AC3, it is unclear whether a change in the kinetics of production and removal of cAMP would explain the actions of the different treatments on STA. To gain insights on the mechanisms that might explain the effects of the different treatments on STA, we developed a computational model of the second messengers and ion channels of the olfactory cilium.

### A quantitative description of olfactory short-term adaptation

EOG signals result from the sensory currents generated by the olfactory cilia of responding OSNs [47]. Thus, we developed a stochastic computational model of the transduction currents of the CNG and CAC channels and the dynamics of their respective ligands in a single cilium to provide a quantitative description of the action of the signaling pathways in the regulation of the levels of STA. The model was implemented stochastically to capture the noise environment of a single olfactory cilium [38], [39], which is a typical property of biological systems containing small number of molecules [48].

**Figure 5:**
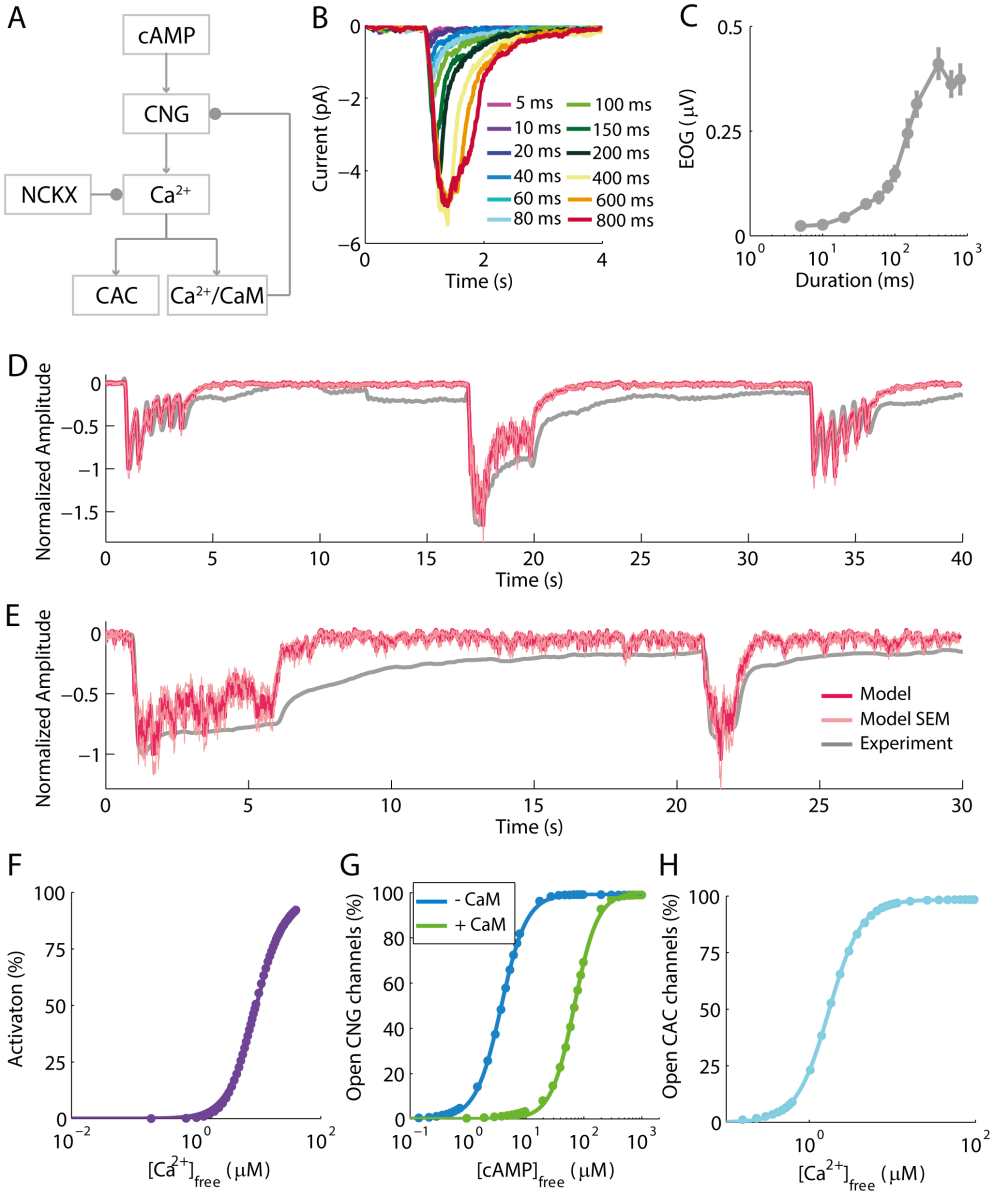
The stochastic model of STA. A: Diagram of the components of the computational model containing the dynamics of cAMP and Ca^2+^ and their actions in the CNG and CAC channels. cAMP opens CNG channels that are permeable to Ca^2+^ that then opens CAC channels. Simultaneously, Ca^2+^ binds to CaM, and the NCKX restores the resting intracellular [Ca^2+^]. In addition, Ca^2+^/CaM binds to CNG channels reducing its affinity by cAMP, which desensitizes these channels. Arrows represent activation and dots represent inactivation. B, C: The computational simulations of the CNG and CAC channels-mediated currents (B) and its respective EOG signals in response (C) to pulses of cAMP of increasing duration reproduced the experimental data shown in Figure 1C and D. D, E: Model response to a more complex stimulation pattern. The model curves correspond to mean results and SEM of 50 single runs of the model. D: Response of the model to pulses composed by slow (2 Hz) and fast (5 Hz) frequencies of cAMP pulses of 100 ms reproduced the experimental EOG response to odorant puffs of the same frequency and duration. E: Result of the model to pulses of cAMP of 5 s and 1 s reproduced the experimental EOG responses to odorant puffs of the same duration. F, G, H: Validation of the isolated components of the model. F: Interaction of Ca^2+^ to CaM. The percentage of activated CaM (CaM totally filled with four Ca^2+^) increases with the concentration of free Ca^2^ and has n_Hill_ = 1.83, and K_1/2_ = 9.25 µM. G: Interaction of CNG channels with cAMP. The percentage of open channels increases with the concentration of free cAMP. The control result shows the percentage of open CNG channels in the absence of Ca^2+^, and the adapted curve shows the percentage of open CNG channels in the presence of saturating Ca^2+^ (20 µM) and CaM (10 µM). The adapted model has an increase in the K_1/2_ for activation of the channels by cAMP with one CaM available per CNG channel. The parameters for fitting the control curve are n_Hill_ = 1.98, and K_1/2_ = 3.743 µM; n_Hill_ = 2.158, and K_1/2_ = 68.49 µM for fitting the adapted curve. H: Interaction of CAC channels with Ca^2+^. The percentage of open CAC channels increases with the concentration of free Ca^2+^. The parameters for fitting the curve are n_Hill_ = 2.21, and K_1/2_ = 1.71 µM.

**Figure 6:**
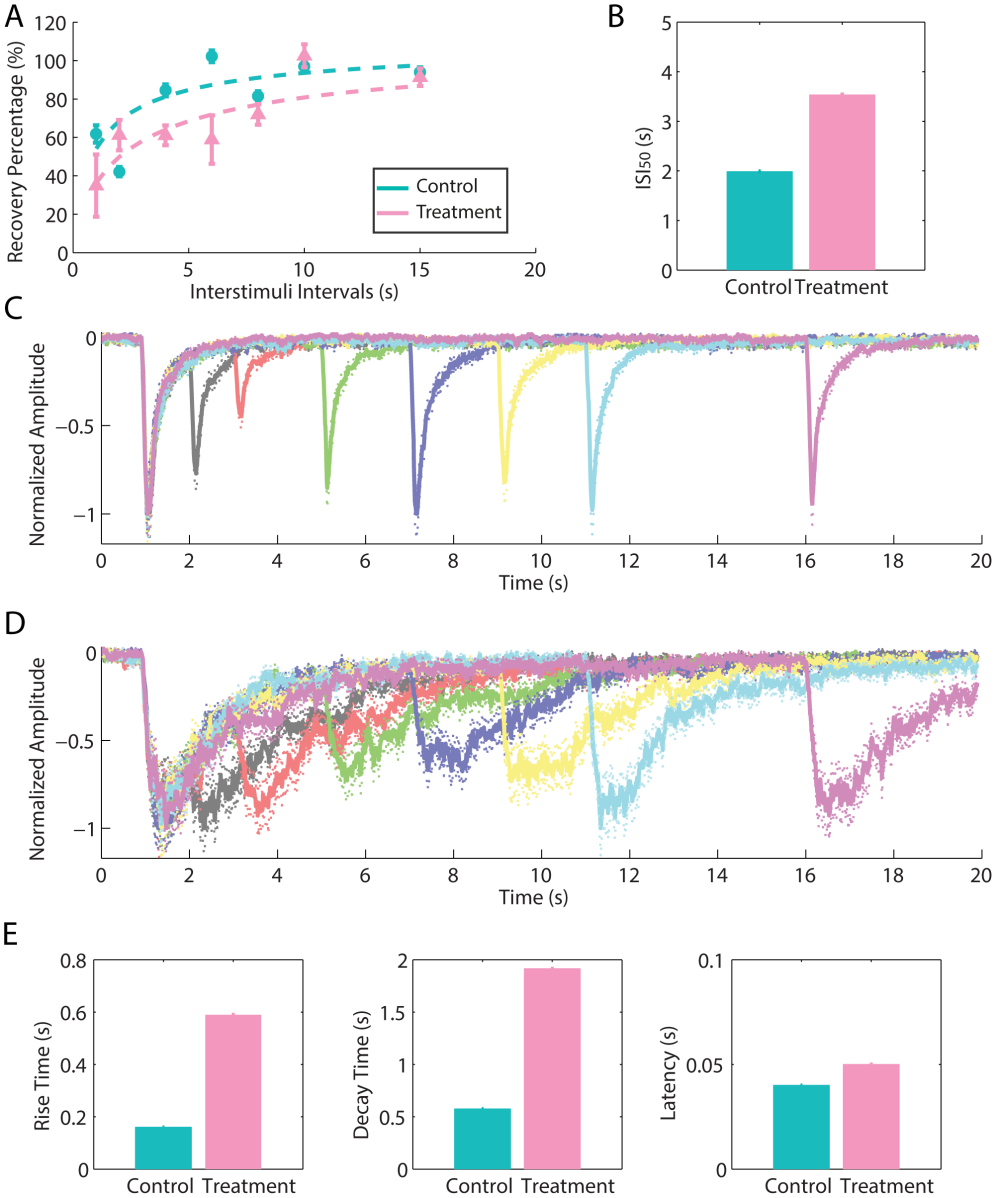
Stochastic modeling of STA. Simulations reproduced the general effects of the pharmacological treatments (IBMX, okadaic acid and monensin). Results are presented as the mean±SEM of 100 single runs of the model. A: Averaged percent of recovery from adaptation for each simulated condition. The interval between paired pulses of cAMP is plotted versus percent of recovery from adaptation. B: ISI for half-maximum recovery from adaptation. C, D: Representative traces of simulated EOG responses during the occurrence of adaptation for different ISIs (1, 2, 4, 6, 8, 10, and 15s). Each trace shows the mean result of 100 stochastic single runs. The standard error is represented by the dotted traces. Simulation of the EOG responses in control condition (C) and treated condition (D). E: Parameters of simulated responses to a single pulse of cAMP.

Although it is well known that Ca^2+^ is required for the occurrence of STA [49], it is unclear how the action of signaling pathways regulating the intensity and time course of cAMP and the Ca^2+^ influx through CNG channels modulate the rise time, decay time, latency and adaptation of sensory responses to odorants. Thus, the model simulated pulses of cAMP that activate CNG channels. The Ca^2+^ influx through these channels rises the intracellular [Ca^2+^] leading to the activation of CAC channels. Moreover, Ca^2+^ binds to CaM, which desensitizes the CNG channels through a decrease in its affinity for cAMP. In addition, NCKX restores the intracellular Ca^2+^ levels. A diagram of the model is shown in Figure 5A. The parameters and reactions used to simulate each one of its components were taken from the literature (see methods). The whole model was validated by comparison with experimental data. Thus, Figure 5B and C show respectively the simulated receptor current and EOG responses of the unitary cilium model to varying durations of input signal, similar to the experimental results in Figure 1C and D. Figure 5D and E show the simulated and experimental responses to bursts of input signal at 2 Hz and 5 Hz (Figure 5D, Figure S4A in File S1) and to prolonged pulses with different duration (5 s and 1 s, Figure 5E, Figure S4B in File S1), and indicate that the model reproduces the experimental data with good accuracy. In addition to the validation of the whole model, we validated its individual components using dose-responses curves (Figures 5F, G and H). These curves were obtained by fitting the Hill equation:
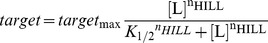
(2)


where *target* refers to the molecule that has been analyzed (CaM, CNG channels or CAC channels), *target_max_* is the maximum activation of the target, n_HILL_ is the Hill coefficient and K_1/2_ is the concentration of the ligand (*L*) required to fully activate half of the total amount of the target. The curves have parameters for the components of the model in accordance with experimental published values (Figures 5F, G and H) [50]–[54]. As a final point, the model simulated brief paired pulses of cAMP separated by multiple ISIs to reproduce the results of STA obtained with EOG recordings (Figure 6).

We simulated the general pharmacological effects of IBMX, okadaic acid and monensin in the EOG signal, which slowed down the kinetics and increased the levels of adaptation. The model presents the typical recovery percentage from adaptation versus ISIs for the control condition, IBMX, okadaic acid and monensin (Figure 6A). Also, the *ISI_50_* (Figure 6B) obtained from the simulated curves reproduced the *ISI_50_* obtained experimentally (Figure 2A, inset). In addition, the model fitted the kinetics of the EOG responses to odorants (Figure 6C, D and E). Simulated effects of IBMX, okadaic acid and monensin also increased the rise time and decay time of EOG responses (Figure 6D and E). The latency of EOG responses to odorants was not simulated explicitly, once it depends on upstream signaling cascades that were not considered in the model, including the binding of odorant to GPCR, the activation of G_αolf_ and AC3. Therefore, the simulated latencies are smaller and represent only the time lag between the release of cAMP and activation of CNG and CAC channels (Figure 6E). In the model, the dynamics of the cAMP signal is determined by its rate of production and hydrolysis in the cilium. To simulate the control condition, paired pulses of free cAMP with duration of 100 ms, amplitude of approximately 6 µM and a rate of hydrolysis of 25 s^−1^ reproduced accurately the results of the control group (Figure 6A, B, C, E). To simulate the effects of the inhibition of PDE, phosphatase and GPCR internalization, we changed the peak amplitude and rate of hydrolysis of cAMP. The amplitude of free cAMP pulses was increased to approximately 15 µM and the hydrolysis decreased to 0.4 s^−1^ to model the effects of IBMX, okadaic acid, and monensin. Consequently, these changes in the dynamics of cAMP prolonged the Ca^2+^ signals, CNG currents and CAC currents (Figure 6C, D, Figure S2 and S3 in File S1), reproducing the experimental data (Figure 2 and 4). The cAMP input used to simulate these effects are provided in the Figure S5 in File S1.

## Discussion

We recorded EOG responses to odorants and used pharmacological inhibitors to study how key enzymes expressed in the olfactory sensory cilia regulate olfactory transduction and adaptation. We used brief paired pulses of odorant stimulation to study the molecular mechanisms of regulation of STA and minimize the occurrence of DS. Our results provide evidences that STA in intact olfactory epithelium is regulated not only by Ca^2+^ acting on CNG channels, but also by other signaling cascades controlling the intracellular levels of cAMP. These results bring innovative evidences about the participation of PKA, PDE and vesicle internalization in the regulation of STA.

We observed a strong effect of IBMX that increased the levels of STA (Figure 2), and this result is consistent with our stochastic simulations that predicted that alterations of the cAMP pulses are required to replicate the effects of IBMX on STA (Figure 6). Breer and colleagues [55] showed that GRK is involved in odorant-induced desensitization of cAMP levels, and inhibition of PKA by Walsh inhibitor or inhibition of GRKs by heparin blocks this process. Dawson and colleagues [26] confirmed the role of GRKs as mediators of this type of odorant-induced desensitization, and demonstrated that disruption of GRK3 genes led to loss of odorant receptor desensitization monitored by the level of cAMP [27]. This process has been shown to involve internalization of GPCRs mediated by a clathrin endocytic pathway that requires the participation of the GTPase dynamin [56]. Other evidences indicate the participation of clathrin pathway during constitutive internalization and recycling of olfactory receptors [57]. Mashukova and colleagues [58] showed that mice pretreated with prolonged odorant stimulation has increased PKA phosphorylation and β-arrestin 2 accumulation in the dendritic knobs of OSNs. Moreover, treating the olfactory epithelium with H89 decreases the accumulation of β-arrestin 2, suggesting that GPCR endocytosis is mediated by a PKA-dependent recruitment of β-arrestin 2 [58]. The phosphorylation of phosducin, a regulator that tightly binds G_βγ_-subunits of G_olf_, catalyzed by PKA reduces its affinity for binding G_βγ_ making it available to anchor GRK3 in the membrane [27], [59]. GRK3 mediates the agonist-dependent phosphorylation and uncoupling of GPCRs from G_αolf_, which potentiates the desensitization observed in the intracellular concentration of cAMP in the olfactory cilia [27]. In addition, EOG responses to odorants, and dissociated cells treated with a dynamin inhibitory peptide (DIP) present a significant loss of adaptation recorded by a gradual decrease in the levels of cytosolic Ca^2+^ in response to repeated odorant stimulation with an ISI around 40 s [58]. However, these studies did not address the role of PKA and dynamin in STA in the intact olfactory epithelium.

**Figure 7:**
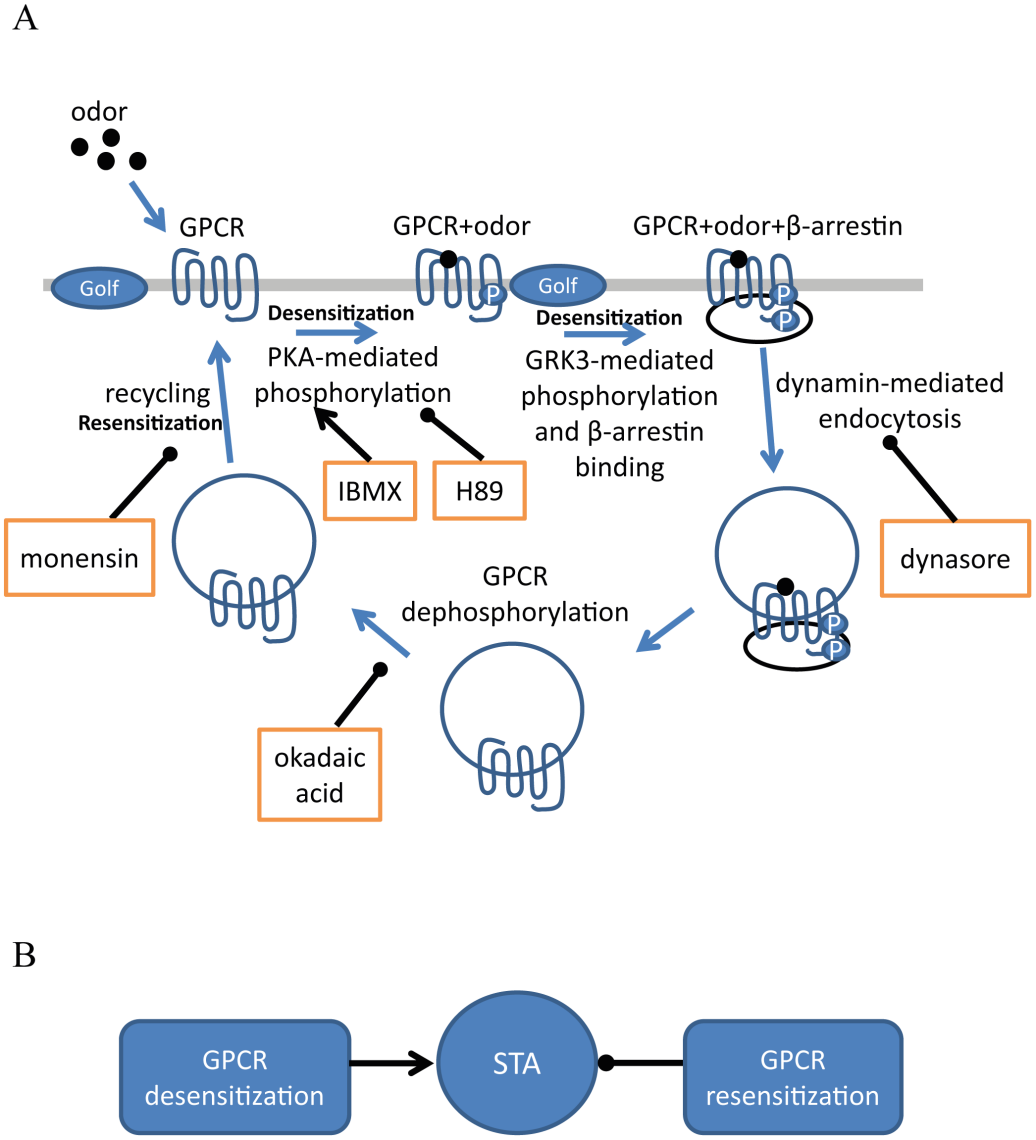
Mechanisms of regulation of STA by GPCR cycling. A: Odorants bind to GPCRs that are phosphorylated by PKA or GRK3 allowing the interaction of the GPCR and β-arrestin. Then, dynamin-mediated endocytosis internalizes the GPCR that can return to the membrane after its dephosphorylation by phosphatases. IBMX increases the levels of cAMP that activate PKA as a consequence of PDE inhibition. H89 inhibits PKA phosphorylation. Dynasore inhibits dynamin. Okadaic acid inhibits phosphatases. Monensin inhibits vesicle recycling. B: Respectively, GPCR desensitization increases and GPCR resensitization decrease the levels of STA.

In our experimental conditions, we did not observe a significant effect of PKA inhibition by H89 and vesicle internalization inhibition by dynasore in the levels of STA (Figure 4). However, we observed an effect of PKA inhibition and vesicle internalization inhibition in the presence of IBMX (Figure 3). These results suggest that a large increase of the cAMP concentration is required to activate PKA and trigger GPCR internalization. IBMX is usually used to increase cAMP levels, as a result of PDE inhibition, and to activate the PKA pathway in cell cultures [60]. Also, IBMX is often used to raise cAMP levels to trigger CNG channel responses in isolated OSNs [61]. Thus, our results suggest that PDE plays a key role in this system by controlling the activation of PKA and GPCR internalization (Figure 7A), and regulating the amplitude and duration of cAMP responses to odorants, and, consequently, the levels of STA (Figure 7B).

Regarding the particular action of PDEs in STA, Boccaccio and colleagues [12] stimulated the OSNs under whole cell path-clamp using caged-cAMP instead of odorants and in the presence of AC3 blockers. They stimulated OSNs using a caged-cAMP analog that is not hydrolyzed by PDEs. These experiments did not demonstrate any difference in STA in comparison to those obtained in the presence of the regular cAMP that is hydrolyzed by PDEs. Therefore, their study suggested that STA is independent of the activity of PDEs [12]. On the other hand, studies performing EOG recordings on mice with disruption of genes expressing PDE in the cilia of OSNs showed attenuated adaptation to repeated stimulation [14]. Differently from Boccaccio and colleagues [12] and Cygnar and Zhao [14], the present results in more intact conditions show that pharmacological inhibition of PDE generates a strong increase in STA measured as a reduction in the peak-to-peak amplitude of the second EOG pulse relative to the first. This result suggests that inhibition of PDE disrupts cAMP degradation in the cilia increasing AC3-dependent cAMP concentration in OSNs. It is likely that this AC3-dependent increases in cAMP concentration did not occur in Boccaccio and colleagues [12] experiments because they only stimulated the OSNs using caged-cAMP instead of odorants and always in the presence of AC3 blockers. Regarding Cygnar and Zhao experiments [14], their PDE knock-out mice had compensatory reduced levels in the expression of AC3, which could explain a reduction of STA.

Since PDE inhibition increases cAMP levels and activates PKA, which facilitates GRK3-dependent phosphorylation of GPCRs allowing its internalization and removal from the membrane [26], presumably, disruption of vesicle internalization through inhibition of PKA phosphorylation or inhibition of dynamin-dependent endocytosis may increase the number of odorant receptors in the ciliary membrane [55], [58], increasing the odorant-induced AC3-dependent cAMP levels, and reversing the effects of IBMX (Figure 3A). On one hand, GPCR phosphorylation by PKA and GRK3 increases internalization, on the other hand, GPCR dephosphorylation results in exocytosis and resensitization [45], [62]. Thus, inhibition of dephosphorylation increases GPCR internalization (Figure 7A) and explains the similar results obtained using okadaic acid, the recycling inhibitor monensin, and IBMX in the levels of STA (Figures 2 and 4).

**Figure 8:**
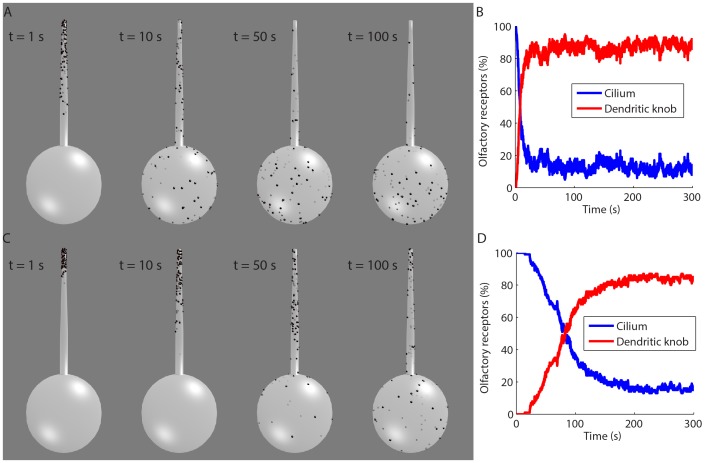
Simulation of the lateral diffusion of olfactory receptors from the membrane of the olfactory cilium to the membrane of the dendritic knob. A: Spatiotemporal distribution of odorant receptors (dots) along the olfactory cilium and dendritic knob for a diffusion coefficient of 0.5 cm^2^.s^−1^. B: Time course of odorant receptors in the olfactory cilium and dendritic knob for the same diffusion coefficient as in A. C: Spatiotemporal distribution of odorant receptors for a diffusion coefficient of 0.05 cm^2^.s^−1^. D: Time course of odorant receptors for the same diffusion coefficient as in C.

In addition to the experimental procedures, we also implemented a stochastic computational model to gain insights on the dynamical properties of the molecular aspects of STA that we have observed. Previous computational models have generated important contributions for the understanding of the mechanisms of olfactory transduction and adaptation in the olfactory cilium. However, they all present limitations that we believe would compromise their use to complement our experimental data. One of the pioneer model of olfactory transduction was constructed to represent the temporal dynamics of transduction currents in the olfactory cilium in response to cAMP and reproduced oscillatory patterns in response to odorants in fish [31]. This model adopted many empirically determined parameter values and simplified CNG and CAC channels to one inactive and one active state driven by the respective steady-state Hill equations of each channel. Later, this model was used to reconstruct EOG oscillations induced by odorant stimulation [32] expressed as a relative and dimensionless value derived from the number of activated CNG and CAC channels of a sub-population of OSNs. In this way, the simulated EOG was driven by a receptor current calculated in a voltage-clamped cilium and derived from a voltage-unclamped membrane potential. However, EOG is likely a local field potential originated from currents flowing from the olfactory cilium and crossing a trans-epithelial bulk resistance [47], [63], [64], rather than originated from a trans-membrane potential. Another model that incorporated the temporal dynamics of odorant-receptor interactions and intracellular transduction events involving cAMP and Ca^2+^ and their effector channel activation and desensitization was used to predict current responses of OSNs to brief and prolonged odorant stimulation [30]. This model reproduced several experimental data of adaptation and desensitization patterns obtained from suction pipette currents recordings in isolated OSNs with unclamped voltage, but using different sets of parameter to simulate each situation. Another model was developed to study the temporal dynamics of the interaction of Ca^2+/^CaM on the CNG channel in response to paired pulse experiments and prolonged pulses, and it was able to capture basic properties of STA and Ca^2+^ oscillations in the cilia [35]. However, this model simulates STA considering an irreversible binding of Ca^2+^/CaM to CNG channels forcing their inactivation, which is unlikely to happen [53], [65]. Recently, a model study of the dynamical transduction events involving receptor, G-protein and AC3 was calibrated to simulated cAMP production rates to complement previous modelling studies in vertebrate cilia [36], but without incorporating the binding and gating events of CNG and CAC channels implicated in olfactory adaptation. Another dynamical model of olfactory transduction was developed to study the basic mechanisms of olfactory adaptation in the olfactory cilia [33], [34]. This model included the kinetics of the CNG channels and the dynamics of cAMP, Ca^2+^, and CaM acting on the desensitization of CNG channels by its interaction Ca^2+^/CaM. Similar to previous models, it reproduces well the macroscopic characteristics of the receptor current using Hill equations and simplified kinetic schemes of negative feedback systems solved deterministically. Although this approach is useful to study the main macroscopic aspects of olfactory transduction and adaptation, it does not allow one to explain how these macroscopic phenomena emerge from the stochastic events happening in the noisy microdomains of the olfactory cilium [38], [39], [66], [67]. Since the signaling pathways regulating olfactory transduction and adaptation is confined in a small cellular compartment [68] and, in consequence, involves low number of molecules [69]–[71], the action and interpretation of the drug effects on the regulation of these events should consider a stochastic approach. In this way, we reproduced the gating and sequential and cooperative binding steps of cAMP in the CNG channels [65] and its desensitization by Ca^2+^/CaM [72] to simulate their population macroscopic currents as an emergent property of the system [53], [73]. The same approach was used for modeling the CAC channels [54], [74]. This model represented successfully the underlying stochastic events occurring in the olfactory cilium and predicted the transduction currents resulting from the intracellular dynamics of cAMP and Ca^2+^ during a protocol of paired-pulse stimulation. Moreover, the model predicted the outcome of EOG signals emerging from these currents in the presence of drugs that interfered in signaling pathways involved in the regulation of GPCR cycling. This model provides a self-consistent framework for predicting both signal and noise components of EOG responses with inferences to single cell electrophysiology data, and produced compelling predictions regarding the regulatory role of GPCR cycling in olfactory transduction and adaptation. In particular, the model predicted how stochastic events drive the variability of single-cell and even single cilium responses. The applications of the model are powerful and certainly go beyond the experiments covered in this work. The model can be used not only to explore the sensitivity of the parameters involved in sensory transduction and adaptation, but also to predict the effects of expression levels of proteins in the cell-to-cell variability in the physiological responses to odorants by randomizing the values of key parameters of the model. In this way, experimentalists can gain a lot from the model. The dynamics of cAMP and Ca^2+^ inside of the nanostructure of the olfactory cilium is difficult to access experimentaly, and future experiments will be required to confirm the predictions of the model by measuring the levels of cAMP in real time in the olfactory cilia of transfected OSNs. Further works can be performed to study the specific sites of endocytosis and visicle recycling in the olfactory cilium. Endocytosis is reported to occur at the ciliary pocket at the base of cilia [75], [76]. Although ciliary pockets have not been found in OSN [77], it does not rule out the possibility that endocytic sites in the pericilliary membrane can regulate the trafficking of GPCRs and play a role in the mechanisms of olfactory reception, since the functional modules used in cilia-related vesicular trafficking are evolutionary conserved [78]. Given the diameter of the olfactory cilium, which is between 100 to 200 nm, and the presence of the axoneme with 9+2 tubulin system [79], small space is left for vesicle endocytosis and exocytosis within the olfactory cilium. In this way, most likely, the dendritic knob is the main site of endocytosis and exocytosis of GPCRs. Menco and colleagues [80], [81] had observed particles in the membranes of olfactory cilia, and plenty of endocytotic vesicles in the dendritic knob surrounding the olfactory cilia [82]. Additionally, proteomic analysis of the olfactory cilium has identified the presence of Rab proteins [83], [84]. Rab proteins are essential for the trafficking of proteins in primary cilium. Generally, the trafficking of proteins to small structures such as the cilia involves the exocytosis from cytosolic vesicles to the plasma membrane in periciliary regions. After the exocytosis, these proteins are transported from periciliary regions to the cilium [85]. However, endocytosis of proteins from the olfactory cilium requires their transport to periciliary regions. Thus, we reconstructed the geometry of the initial segment of the olfactory cilium and dendritic knob to verify the time window of the diffusion of receptor proteins from the olfactory cilium to the dendritic knob. Computational simulations of the lateral diffusion of olfactory receptors in the membrane of the olfactory cilium towards the membrane of the dendritic knob, considering diffusion constants in a range observed for metabotropic receptors [86], indicated that this process occurs in the time window of seconds (Figure 8A, B) to minutes (Figure 8C, D), which is consistent with our experimental conditions.

Olfactory transduction is a non-linear process controlled by a network of signaling pathways forming multiple negative-feedback systems [11], [14]–[17], [26], and it is not straightforward to predict how desensitization observed in the levels of GPCR and cAMP is involved in STA at the level of the ionic currents. Our results showed an effect of IBMX, monensin and okadaic acid in the intact olfactory epithelium and revealed the roles of vesicle internalization and recycling in the levels of STA regulated by negative feedback systems (Figure 7A and B). These experimental results are linked to our modeling results since both transduction currents and EOG signals result from elevations in the cAMP concentration that activate CNG channels. The dynamics of cAMP is a complex process that is regulated by the equilibrium between its production by AC3, which is regulated by GPCR cycling, and its diffusion and hydrolysis by PDE.

The presence of desensitization upstream from cAMP production has been reported previously with electrophysiological experiments [87], [88], but the molecular mechanism of this process is not resolved. The present data provides evidence that this process may involve GPCR desensitization and recycling. Presumably, IBMX increases GPCR desensitization, and monensin and okadaic acid reduces GPCR resensitization. The consequence of this process is a decreased number of GPCRs in the membrane, resulting in a lower activation of AC3 and a reduced production rate of cAMP. The stochastic kinetic model described in this work predicted that a reduction in the cAMP hydrolysis rate leads to an intense cAMP signal that increases the levels of STA (Figure S5B in File S1). This reduced cAMP hydrolysis rate might result from a lower increase in intracellular Ca^2+^ concentration (Figure S3F in File S1) that would prevent the activation of the Ca^2^/CaM-dependent PDE1C in the cilium [89]. Our model predicted that this Ca^2+^ signal is reduced but prolonged, which favored the occurrent of Ca^2+^/CaM-dependent CNG channel desensitization (Figure S3F in File S1). In control conditions, there is a different scenario. The normal levels of GPCR in the membrane lead to a strong production rate of cAMP. However, under this condition, there is also a high hydrolysis rate of cAMP, which presumably results from the intense increase in Ca^2+^ signals (Figure S2F in File S1) and strong activation of PDE1C. This activation of PDE1C causes a reduced elevation in the free cAMP (Figure S5A in File S1), which promotes the control level of STA (Figure S2F in File S1). All together, our results suggest that the magnitude of STA and recovery times from adaptation are regulated by the kinetics of cAMP signals emerging from the number of GPCR in the membrane of the OSNs. However, it is important to note that additional molecular processes also could account for the results described in this work. Several signaling networks rely on the inhibition of phosphatases activity as a mechanism contributing to long-lasting processes [90]. For example, in neurons from the striatum, PKA phosphorylates a molecule termed dopamine-and cAMP-regulated neuronal phosphoprotein (DARPP-32) converting it to a potent inhibitor of the ubiquitous protein phosphatase (PP1) [91]. This process is involved in the regulation of several targets, including ionic channels and the Na^+^/K^+^ ATPase. It is not known whether DARPP-32 or some analogous molecule [90] is expressed in OSNs, but we cannot exclude that a possible inhibition of PP1 by PKA can contribute to strength STA consistently with our results obtained by blocking the protein phosphatases with okadaic acid. Furthermore, we cannot rule out that signaling pathways involved with DS to odorants might also participate in the reduction of the intensity of EOG responses to paired pulses of odorants. There are evidences that DS is mediated by the inhibitory phosphorylation of AC3 by Ca^2+^/CaM kinase II (CaMKII) [28], [92], but a mutation on the residue responsible for CaMKII phosphorylation of AC3 in mice did not attenuate olfactory responses [93]. Thus, it is unclear whether the Ca^2+^ influx triggered by cAMP acts selectively on CNG, CaMKII or PDE to disrupt transiently the action of cAMP with distinct onset and recovery times or whether the occurrence of STA and DS involve a certain level of overlap of signaling pathways.

In conclusion, we characterized STA in olfactory responses, including downstream effectors and GPCR cycling, and achieved insights into the various signaling pathways regulating the levels of STA in the olfactory epithelium. Although the participation of DS and DARPP-32 cannot be completely excluded in our experimental conditions, it is clear from our work that, even though STA can be explained by a general mechanism involving the reduction in the affinity of cAMP for CNG channels associated to Ca^2+^/CaM, this process can be controlled and modulated by signaling pathways upstream to it, which allows the system to present a larger regulatory capacity than previously considered. These conclusions may have consequences not only for the functioning of olfactory signaling pathways but also for other regulatory systems in the brain [34], [94], as they reveal the participation of mechanisms of trafficking of metabotropic receptors in the regulation of ion channels in neurons.

## Materials and Methods

All procedures were approved by the Institutional Animal Care and Use Committee (IACUC) of the Fundação para a Ciência e a Tecnologia (FCT). All efforts were taken to comply with the 3Rs (Reduction, Replacement, and Refinement). Reduction in the number of laboratory animals was performed by using both the ipsilateral and contralateral olfactory epithelium. The other parts of the brain were used in other experiments conducted in the lab as another effort to reduce the use of laboratory animals. We have developed a publicly available computational model that can be used as a replacement for experiments using animals. Refinement to minimize potential pain, suffering and distress were taken by using appropriate anesthetics.

### Animal surgery

The animals were handled according to European Community guidelines and Portuguese law concerning animal care. The experiments were performed in 4 to 7 weeks old male Wistar rats. Rats were anesthetized with isoflurene before decapitation. The head was put over ice and the skin and lower jaw were removed. The remaining skull was immersed in ringer solution at 10°C, and cut sagitally. The nasal septum was removed to expose the intact turbinates with the olfactory epithelium and each side of the skull containing olfactory epithelium was maintained at room temperature.

### Solutions

The normal ringer solution was used in the recording pipette and perfusion of drugs in the olfactory epithelium. The concentration used of each component of the ringer solution was NaCl (140 mM), KCl (5 mM), Hepes (10 mM), Mg.Cl_2_ (1 mM), CaCl_2_ (1 mM), Napyruvate (1 mM), D-Glucose (10 mM). The pH 7.4 was adjusted with NaOH. Fresh solutions were made weekly and maintained refrigerated.

### Data acquisition system

EOG recording was performed with a Patch Clamp Amplifier L/M EPC 7 (Darmstadt, Germany) connected to a Digidata 1140A digitizer (Axon Instruments) controlled by a PC microcomputer running the software pCLAMP 10 (Axon Instruments).

### Odorant stimulation

Odorant responses were elicited by a mixture of Acetophenone (Sigma Aldrich) and Cineole (Sigma Aldrich) diluted to 50 mM in distilled water. The vapor of odorant solution was puffed to the olfactory epithelium by releasing a flow of purified Nitrogen at 20 psi through an odorant bottle connected to an air delivery tube placed at 1 cm from the olfactory epithelium. The puff duration was regulated by a Pneumatic Picopump PV 820 (World Precision Instruments - WPI) triggered by a S48 square pulse electrical stimulator (Grass Technologies) controlled by software using a custom made protocol developed in pCLAMP 10 (Axon Instruments). Odorant responses were elicited by paired-pulses of 100 ms separated by ISIs of 1, 2, 4, 6, 8, 10 and 15 s. We performed 3 trials per condition separated by 1 min intervals.

### Drugs

All drugs were purchased from Sigma Aldrich. We use a variety of cell-permeable inhibitors to disrupt the catalytic activity of enzymes expressed in the cilia of OSNs as follows: the selective and potent inhibitor of PKA N-[2-(p-Bromocinnamylamino)ethyl]-5-isoquinolinesulfonamide (H-89) [95]; the non-specific inhibitor of PDEs dihydrochloride hydrate (3-isobutyl-1-methylxanthine (IBMX) [96]; the non-competitive inhibitor of dynamin GTPase activity dynasore hydrate [46], [97]; okadaic acid, inhibitor of protein phosphatases 1, 2A and 2B [98]; and monensin sodium salt, a carboxylic ionophore that interrupts GPCR recycling [42].

We screened the olfactory epithelium with concentrations ranging from two- to ten-fold the half maximal inhibitory concentration (IC_50_) to disrupt the activity of each enzyme [99]. The concentrations used were: H-89 at 20 µM, IBMX at 190 µM, dynasore at 25 µM, okadaic acid at 10 µM, and monensin at 10 µM. Aliquots were prepared in DMSO and maintained at −20°C. Aliquots were diluted to the proper concentrations in normal ringer solution in the day of the experiment and perfused on the top of the olfactory epithelium at 500 µL/min during 20 min using a peristaltic pump Minipuls (Gilson). EOG recordings started 60 min after perfusion. The control recordings were performed 60 min after 20 min of perfusion of vehicle (ringer + DMSO). We could not conduct recordings during the perfusion because the EOG signal is lost in the wet epithelium.

Our experimental and modeling results provide evidences that the observed effects generated by IBMX, okadaic acid and dynasore result from increased levels of cAMP in the olfactory epithelium. In this way, we used cAMP to mimic the general effects of these pharmacological treatments, and washed out the drug to reverse the effects of increasing levels of cAMP. The tissue was perfused during 20 min with a solution of normal ringer, 0.02% DMSO, and 500 µM of N^6^,2′-O-Dibutyryladenosine 3′,5′-cyclic monophosphate sodium salt (Sigma-Aldrich), a cell-permeable cAMP analog. Then, the olfactory epithelium was washed during 20 min with a solution of normal ringer and 0.02% DMSO. All recordings started 60 min after the perfusion. We did not observe significant differences between the L, RT and DT of the EOG signal of the treated and the washed groups (Table S1 in File S1).

### EOG recording

EOG recordings were performed at room temperature. A conductor wire immersed in ringer solution was used as reference. Glass borosilicate electrodes of 3 to 5 MΩ were made by heating and pulling GC150F-10 borosilicate glass capillaries (Harvard Apparatus) using a PC-10 puller (Narishige). Recording electrodes were filled with normal ringer solution, and the tip of the electrode was positioned on the top of the olfactory epithelium by a SM-1 micromanipulator (Luigs & Neumann). We targeted the turbinates 2 and 3 that were kept humidified by a constant humidified air flow of 1.16 L/min (Eco-air 9800).

### Data analysis

The analysis was performed using Matlab (Mathworks). Prior analysis EOG signals were low-pass filtered at 10 Hz with a fifth order Butterworth digital filter. Standard parameters of the EOG analysis (the response amplitude, latency, rise time, and decay time) were calculated for each single odorant response [100].

The response amplitude was defined as the peak-to-edge amplitude, which was calculated from the difference between the voltage at the peak of the response and the voltage preceding the stimulus. The latency was considered the time lapse between the administration of the stimulus and the beginning of the response at 1% of the peak amplitude. The rise time was defined as the time lapse between the start of the response (1% of the peak amplitude) and the peak. The decay time was determined by fitting the decay phase of the response using a single exponential equation.

The peak amplitude of the EOG responses (Figure S6 in File S1) was normalized prior to analysis, and the latency, rise time, and decay time were compared among pharmacological treatments for statistical differences. The ratio between the second and the first amplitude of response to paired pulses of odorants was calculated for the STA analysis. The ISI for half-maximum recovery from adaptation (*ISI_50_*) was calculated for each treatment.

### Statistics

Data are given as mean ± standard error of the mean (SEM). Statistical analyses for significant differences were performed using the Statistica software (StatSoft, Inc.) and the Matlab Statistics Toolbox (The Mathworks, Inc., Natick, MA). Statistical significance was assessed using one-way analysis of variance (ANOVA) followed by Fisher's Least Significant Difference (LSD) or Tukey's Honestly Significant Difference (HSD) *post hoc* tests, where p<0.05 was considered statistically significant. Fisher's LSD was used in the cases that needed a more sensitive test for multiple comparisons.

## Model

The computational model (source code available at https://senselab.med.yale.edu/modeldb/ShowModel.asp?model=151686, password = 1416) of olfactory adaptation was constructed using Copasi [101], a software that allows stochastic and deterministic solutions of kinetic reactions. Simulations were computed in an AMD Opteron processor 6168×18 running Condor-COPASI, a high-throughput computing environment to run Copasi simulations in parallel [102]. Deterministic simulation solutions for the steady-state validation of the isolated components of the model were solved using the method LSODA that calculates the time course by automatically selecting between non-stiff and stiff methods [101]. Stochastic simulations were performed using the Gillespie direct method implemented in Copasi [101].

The mammalian olfactory cilium is approximately 15 to 50 µm in length and is divided into a proximal (300 nm in diameter) and a distal segment that tapers towards its tip to about 100 nm in diameter [82]. Therefore, a single cilium has an approximate volume of 1 fL based on this geometrical structure. The reactions between intracellular ligands and ion channels located on the surface of the cilium happen in the subvolume just underneath of the membrane. In consequence, we implemented these reactions in a compartment model of 0.2 fL. The Ca^2+^ diffusion between this subvolume and the remaining cilium was modeled using first order reactions with diffusional rate constants scaled for each volume [103]. The model includes the interaction of Ca^2+^ with CaM, the activation of CNG channels by cAMP [65], their desensitization by interactions with Ca^2+^/CaM [72], the activation of CAC channels by Ca^2+^ [54], [74], [84], and the extrusion of Ca^2+^ by NCKX [16], [17] (Figure 5A). The model was stimulated by pulses of cAMP that activate CNG channels. Consequently, the Ca^2+^ influx through the open CNG channels rises the intracellular [Ca^2+^] that activate CAC channels. In addition, adaptation has been incorporated in the model through the association of Ca^2+^/CaM with CNG, which promotes a decrease in the affinity of CNG for cAMP [15], [104]. Each component of the model was simulated according to the reactions and parameters described in the literature. The number of copies of each component of the model is listed in Table 1.

**Table 1:** Initial concentrations or number of copies of the components of the model.

Identity	Number of copies/Concentration	Reference
Ca^2+^	40 nM	[144]–[146]
cAMP	0.3 µM	[138]
CaM	5 µM	[11]
CNG	100 molecules (8 channels/µm^ 2^)	[54]
CAC	750 molecules (62 channels/µm^ 2^)	[54]
NCKX	∼1873 molecules	This paper

### Interaction between CaM and Ca^2+^

**Figure 9:**
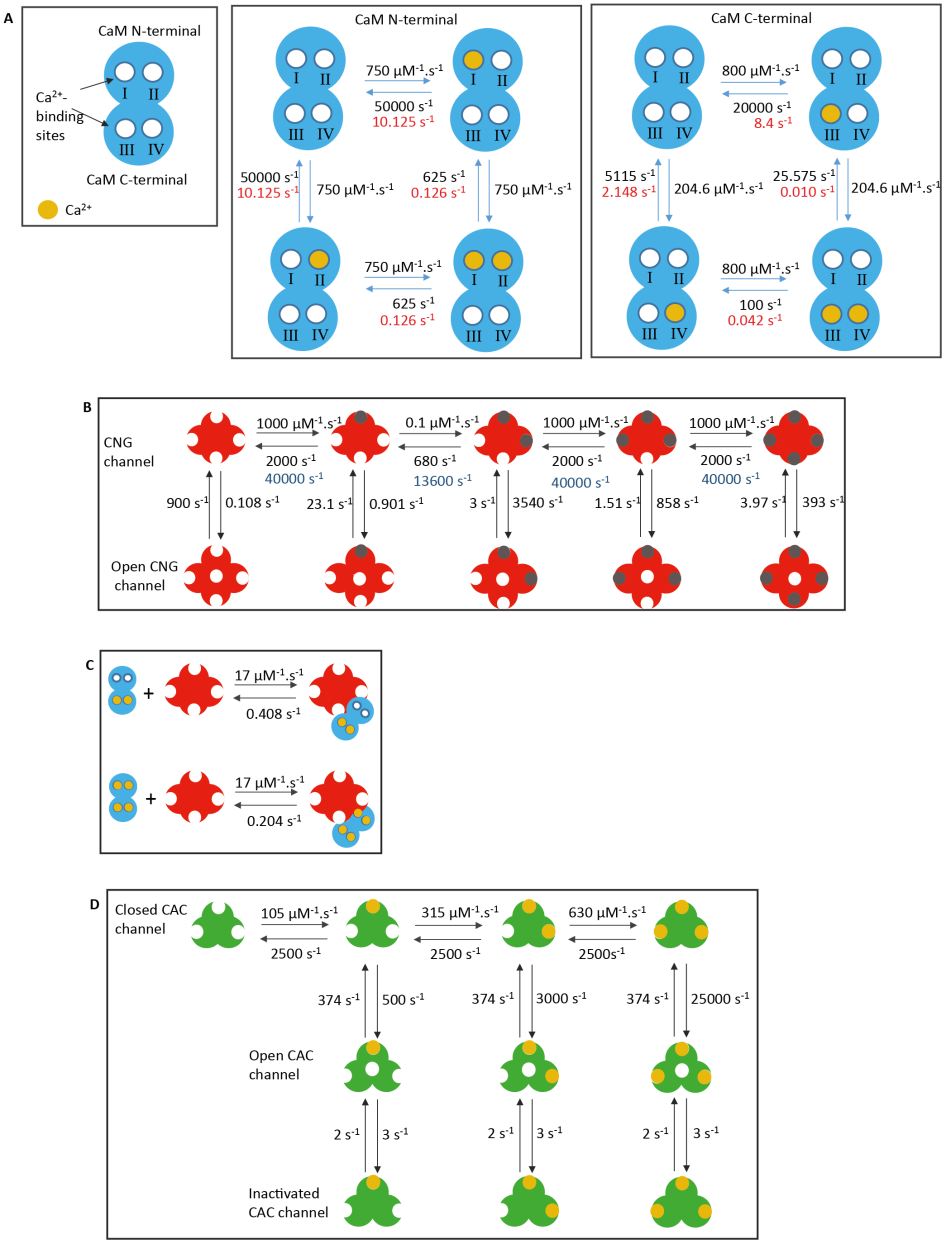
Kinetic schemes and parameters used in the model. A: Binding of Ca^2+^ with CaM. The backward rate constants of Ca^2+^ with CaM are changed in the presence of CNG channels (red). B: Binding of cAMP (gray) with the CNG channels. C: The model of interaction between CNG channels and Ca^2+^/CaM considered that CNG channels can associate to CaM with less than four Ca^2+^. However, the interaction between CNG channels and CaM with one or two Ca^2+^ has a weaker affinity than the interaction between CNG channels and CaM with three or four Ca^2+^. Ca^2+^/CaM can bind to open and closed CNG channels with the same affinities. D: Binding of Ca^2+^ to CAC channels.

In the olfactory cilia, CaM has a pivotal role regulating the activity of many molecules involved in different aspects of the olfactory transduction and adaptation [105]. Structurally, CaM consists of two globular domains (the carboxyl-terminal and amino-terminal domain) [106], [107], each one containing a pair of Ca^2+^-binding motifs called EF-hands that bind Ca^2+^ sequentially with positive cooperativity [108]–[110]. The Ca^2+^-binding sites located at the CaM amino-terminal are termed I and II, and those located at the carboxyl-terminal are termed III and IV (Figure 9A). The binding of Ca^2+^ to CaM promotes a conformation change of each EF-hand pair domain that leads to the exposure of hydrophobic pockets that provide interaction sites for targets molecules [111]–[113]. Each CaM domain can switch from closed to open conformation after the binding of a single Ca^2+^ [114]. However, the association of two ions to each domain is important for stabilizing this open conformation [111], [114]–[116]. To define the parameters for the interaction of Ca^2+^ to CaM used in the model, it was considered that each CaM domain has two macroscopic association constants, K_1_ and K_2_. K_1_ is the sum of the microscopic equilibrium constants for homotropic cooperativity considering that individual Ca^2+^-binding sites of either domain are occupied sequentially [117]–[120]:
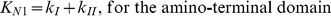
(3)

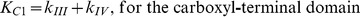
(4)where *k_I_*, and *k_II_*, *k_III_*, and *k_IV_*, are the microscopic equilibrium constants of the Ca^2+^-binding site I, II, III, and IV, respectively. Assuming that the affinity for either site of a given CaM domain is equivalent, the values of *k_I_*/*k_II_* (0.015 µM^−1^), *k_III_*/*k_IV_* (0.04 µM^−1^) used to calculate the rate constants of the model were estimated from the literature [117], [118], [121]. Thus, the forward rate constants (k_f_) used to simulate the binding of the first Ca^2+^ to either Ca^2+^-binding sites of both amino- and carboxyl-terminal were calculated using the microscopic equilibrium constants presented above and backward rate constants (k_b_) estimated from the literature [113], [114], [122], [123]. The model considered that the bind of the first Ca^2+^ to CaM occurs in any one of its four Ca^2+^ -binding sites. The cooperativity observed for the binding of a second Ca^2+^ to either CaM globular domain determines that [118], [120]:
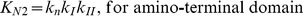
(5)

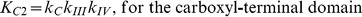
(6)where *k_n_* and *k_c_* are the intradomain cooperative constants for the binding of the second Ca^2+^ to the amino and carboxyl-terminal respectively [117]–[120]. No interdomain cooperativity was considered in the model as isolated CaM domains exhibit binding properties similar to the corresponding domains in intact CaM [108], [124].

The values of *k_n_* (80 µM^−1^), and *k_c_* (200 µM^−1^) used to calculate the rate constants of the model were estimated from the literature [117]. To estimate the rate constants for the binding of a second Ca^2+^ to each one of CaM globular domains, the values of k_f_ were kept unchanged as their values are already consistent with diffusion-limited reactions. The k_b_s were calculated considering the microscopic equilibrium constants for the binding of Ca^2+^ to a vacant site of a given domain when the other site is filled. The backward rate constants obtained are within the range of values observed experimentally [123]–[126]. The parameters and reactions used in the model are presented in Figure 9A.

### Interaction of Ca^2+^/CaM with its targets

Following the implementation of the binding of Ca^2+^ to CaM, we simulate its interaction with CNG channels and the interaction between CNG channels and cAMP. Olfactory CNG channels can bind up to four molecules of cAMP [53], [65]. However, there is no kinetic scheme available for the gating of native olfactory CNG channels. Consequently, we simulated the gating of CNG channels using a kinetic scheme developed for homotetrameric CNGα2 channels [127], [128], which captures many of the properties observed for the heterotetrameric CNG channels [53], [65]. But some parameters used in the original scheme had to be changed to incorporate the apparent affinity observed for the interaction between cAMP and the heterotetrameric CNG that is higher in comparison to the affinity observed for homotetrameric CNGα2 channels [65]. The interaction between cAMP and the four CNG channel subunits is sequential [53]. Three subunits interact with cAMP with high affinity. These subunits are the first, third and fourth to interact with cAMP. The second subunit to be filled presents an association constant for cAMP that is smaller than the others [127], [128]. Once the second molecule of cAMP is bound, there is a switch from low to high open probability in the channel [128], [129]. Thus, the binding of additional cAMP molecules provides a further stabilization to the open state of the channel [53]. A representation of the kinetic model implemented to simulate CNG channels and the parameters used are shown in Figure 9B.

Once open, olfactory CNG channels allow the influx of Ca^2+^ to the cilia [130], which causes the fast olfactory adaptation. Adaptation in OSNs is a Ca^2+^-dependent process that involves the high affinity binding of Ca^2+^/CaM to the CNG channels, which promotes a decrease in their affinity for cAMP [15], [104]. The olfactory adaptation mediated by the association of CNG channels with Ca^2+^/CaM has a 1:1 stoichiometry and involves both domains of CaM associated with Ca^2+^ [72]. Therefore, we assumed that the decrease in the affinity of cAMP for CNG is mediated by the binding of these channels to CaM filled with three or four Ca^2+^. The assumption that CaM associated to three and not only four Ca^2+^ causes a decrease in CNG affinity for cAMP was based on the fact that a single Ca^2+^ can promote the switch in the conformation of a CaM domain from closed to open [114], leading to the exposure of residues of interaction [112]. However, the species of CaM bound with pairs of Ca^2+^ are more likely to occur in the model because of the intradomain cooperativity for the binding of Ca^2+^ to CaM. Following experimental data, we considered that the association of CaM bound to three or four Ca^2+^ to CNG channels leads to olfactory adaptation through an increase of 20-fold in the K_1/2_ of CNG channels for cAMP [15], [104], [131]. To simulate this process, we kept the rate constants for the binding (k_f_) of cAMP unchanged and altered the rate constants for the cAMP unbinding (k_b_) (Figure 9B). Thus, the values of k_b_ for the unbinding of cAMP to CNG channels associated to CaM with three or four Ca^2+^ were increased 20-fold to tune the model. At low [Ca^2+^], CNG channels can interact with CaM partially loaded with Ca^2+^, but such interaction does not promote adaptation. The coupling between CNG channels and partially loaded CaM at rest [Ca^2+^] has been shown to be an important factor to allow the system to exhibit fast feedback modulation when the ciliary [Ca^2+^] rises [53], [72]. Consequently, the model of interaction between CaM and CNG channels considered that CaM bound to one or two Ca^2+^ can associate to CNG channels without promoting any alteration in its affinity for cAMP. We assumed a K_D_ for the association of Ca^2+^/CaM to CNG channels of 12 nM [53], [104], and a smaller affinity for the interaction of CNG channels with partially loaded CaM. Thus, the associations between CNG channels and CaM filled with one or two Ca^2+^ where simulated with a k_f_ taken from the literature [132], and a k_b_ calculated considering a K_D_ of 24 nmol.L^−1^ (Figure 9C). For the interaction between CNG channels and CaM bound to three or four Ca^2+^, we kept the k_f_ unchanged and recalculated k_b_ using a K_D_ of 12 nM (Figure 9C) [53], [104]. No difference in the dissociation constant for the interaction between Ca^2+^/CaM and closed or open CNG channels was considered in the model, as the rate for the Ca^2+^-modulation of the channel is independent of its open probability [132]. The association of CaM to CNG channels, similarly to what is observed for other CaM targets [50], [125], [126], increases its affinity for Ca^2+^ [72]. To recalculate the rates of interaction between Ca^2+^ and CaM associated to CNG channels, we kept the k_f_ unchanged (Figure 9A) and recalculate the k_b_s according to equations 3–6 considering a macroscopic K_D_ of 21 nM and 27 nM for the binding of Ca^2+^ to the Ca^2+^-binding sites located at the carboxyl- and amino-terminal, respectively [53].

### Other components of the model

A second species of the model that regulates the intracellular levels of Ca^2+^ is NCKX, which is a bidirectional transporter that exchange Na^+^/Ca^2+^+K^+^ with stoichiometry of 4 Na^+^:1 Ca^2+^ + 1 K^+^ [133], [134]. The main function of NCKX exchangers is to extrude Ca^2+^ from the cytoplasm to the exterior of the cell, a process that is observed in the OSNs [16], [22]. Little is known about NCKX modulation and regulation [133], therefore, its activity was simulated using only catalytic reactions. The transport of Na^+^ and K^+^ were not implemented explicitly. The efflux of Ca^2+^ was simulated as a non-conservative process, and extracellular Ca^2+^ was not modeled explicitly. The reactions that simulates Ca^2+^ extrusion is given as follows:
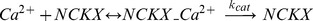
where k_f_ = 250 µM^−1^.s^−1^ and k_b_ = 100 s^−1^ are the rate constants of binding and unbinding of the complex NCKX_Ca^2+^, and k_cat_ = 2400 s^−1^ is the catalytic rate constant.

To counterbalance the activity of NCKX, the model has a non-specific Ca^2+^-leak that pumps Ca^2+^ to the cytoplasm.

To simulate the concentration of cAMP at rest we assumed a basal rate of cAMP production and degradation, which were changed to simulate the effects of the pharmacological treatments tested.

The second type of ionic channel included in the model is the CAC channel. To simulate the CAC channels, we used a kinetic scheme based on a previously proposed model [74]. The rate constants of the CAC channel model were tuned to simulate the fast activation and deactivation kinetics of Anoctamin 2 (ANO2) [135], which is the main constituent of the CAC channels expressed in OSNs [136]. An inactivating kinetic state was coupled to each open state to incorporate the intrinsic CAC channel-mediated current decline observed in native OSNs [54]. The kinetic parameters were adjusted to fit the dose-response curve of the channel accordingly with data obtained from rat native OSNs [54]. The reactions and parameters used in the model of CAC channels are shown in Figure 9D.

### CNG and CAC channels-mediated currents

We considered in the model a total of 100 CNG channel and 750 CAC channels based in the density of these channels in the membrane of rat OSNs [54]. Then, we computed the discrete number of open channels for each time step. The CNG and CAC channels-mediated currents were calculated bearing in mind the number of open channels, the unitary conductance of each channel, the reversal potential of each channel, and the membrane potential of the olfactory cilium. We assumed a CNG channel and CAC channel unitary conductance of 0.56 pS [67] and 1.27 pS [54], respectively, and a reversal potential at 0 mV for each channel [54], [137], [138]. The currents were calculated under voltage clamp with a holding potential of −65 mV, and we adopted a flux of 78000 Ca^2+^/s per single open CNG channel [54].

### EOG responses

The EOG is considered the trans-epithelial voltage resulting from depolarizing receptor currents from the olfactory cilia flowing through the extracellular space [63], [139]. Since the origin of EOG signals is linked to the transduction currents in the cilia of a population of odorant responding OSNs in the field of the recording electrode [47], [100], [139], then the EOG responses were estimated through the summated CNG and CAC channels-mediated current responses from a simulated olfactory cilium multiplied by the bulk epithelium resistance of 75 KΩ [63].

Two main assumptions had to be made. Firstly, we adopted a voltage clamp condition to simulate the transduction currents (CNG+CAC channels-mediated currents), although OSNs are not voltage-clamped during EOG responses. This assumption was considered because most of the experiments available in the literature reporting current responses and odorant adaptation in OSNs have been performed under voltage clamp [11], [87], [138], [140]. In addition, COPASI has a restriction to solve stochastically global quantities defined as ordinary differential equations that are required to model a dynamical membrane potential. Thus, we assumed that non-linear effects of membrane depolarization on the transduction currents would not alter significantly the levels of odorant adaptation in this system. Secondly, we simulated current responses of the olfactory cilium to pulses of cAMP instead of odorants. This simplification was based on the fact that experiments under voltage-clamp that stimulate OSNs directly through uncaging of cAMP accurately mimicked the current responses to odorant puffs [11], [87], [140].

Our simulation results estimated that single cilium EOG responses are in the order of microvolts, suggesting that population EOG responses in millivolts emerge from the synchronized olfactory receptor currents of thousands of olfactory cilia located in the field of the recording electrode.

The pharmacological experiments were reproduced by changing the cAMP input of the model to replicate the experimental EOG output and predict the effects of the pharmacological treatment in the dynamics of the cAMP intracellular concentration. This approach relies on evidences that phosphorylation and disruptions of GPCR internalization have strong effects on the dynamics of cAMP [25]–[27], [55], [59], [141]. In this way, we were able to interpret the downstream effects of the interference in vesicle recycling and GPCR internalization in the production of cAMP by simulating their effects on the activation and desensitization of CNG channels that generate the levels of olfactory adaptation observed in the EOG signals.

### Computational model of olfactory receptor diffusion

The model of olfactory receptor diffusion was developed in Monte Carlo Cell (MCell) [142], [143], which is a tool for particle based quantitative modeling of stochastic reaction-diffusion events in 3-dimensional (3-D) environments. The 3-D approach of MCell comprises the structural representation of the subcellular environment considered and the movements of mobile species. The mesh structure of the dendritic knob and basal segment of the olfactory cilium used to develop a diffusion simulation were constructed using CellBlender. The mammalian olfactory cilium is approximately 15 to 50 µm in length and is divided into a proximal and a distal segment. The proximal segment is shorter and ticker, and is connected to the dendritic knob. The distal segment tapers to approximately 100 nm in diameter [82]. The dendritic knob was represented with a sphere of 2 µm in diameter connected to the base of a cylindrical olfactory cilium with a proximal segment of 3 µm and 200 nm in diameter tapering to 100 nm along the beginning of the distal segment with 3 µm of length. MCell considers that individual molecules move through random walks, which reproduce the displacements that correspond to Brownian motion. We assumed that olfactory receptors can diffuse freely within the olfactory cilium and dendritic knob membranes. Consequently, a corresponding diffusion coefficient was defined for the olfactory receptors in the model. We used a fast diffusion coefficient of 0.5 cm^2^.s^−1^ and a slow diffusion coefficient of 0.05 cm^2^.s^−1^ based on the diffusion coeficients observed for metabotropic receptors [86]. We released 100 olfactory receptors on the tip of the cilium and quantified the number of receptors diffusing from the cilium to the dendritic knob for each time step.

## Supporting Information

File S1 **Supplementary material.**
(PDF)
