# Supplementary material for: Mechanisms of Regulation of Olfactory Transduction and Adaptation in the Olfactory Cilium
Source: PLoS One. 2014 Aug 21;9(8):e105531. doi: 10.1371/journal.pone.0105531 (PMC4140790; doi:10.1371/journal.pone.0105531)
Supplement: File S1 — Supplementary material. (PDF) [file pone.0105531.s001.pdf]

Supplementary Material

The experiments to obtain the dose-response curves of EOG responses to increasing durations of odorants were performed with a mixture of acetophenone and cineole diluted to 50 mM in water (Figure S1). The recordings were performed under control condition with the normal ringer solution. Paired pulses of odorants separated by an interstimuli interval (ISI) of 4 s were puffed with the duration of 5, 10, 20, 40, 60, 80, 100, 150, 200 and 400 ms in the olfactory epithelium. Three repetitions were performed per condition and the mean EOG responses were analyzed using the software Matlab. The amplitude of the mean EOG responses was normalized to the amplitude of maximum response for comparison between conditions. The amplitude of EOG responses to the first pulse, the non-adapted response (red), was compared with the amplitude of EOG responses to the second pulse, the adapted response (blue). The dose-response relation was fitted with the Hill equation (1) with the Curve Fitting Tool (cftool) in Matlab, using the method of nonlinear least squares with the algorithm of trust-region:

$$Response = \frac{EOG_{max}(Dose^{nHILL})}{({K_{1/2}}^{nHILL} + Dose^{nHILL})} \quad (1)$$

The *Dose* stands for the duration of odorant stimulus, and the *Response* stands for the amplitude of EOG response. Fitting of Hill equation was performed with a 95% confidence interval to obtain the parameters for the dose of stimulus that generates the half-maximum response ($K_{1/2}$), the Hill coefficient (n_{HILL}), and the maximum amplitude of EOG response (EOG_{max}). Comparison between the adapted and non-adapted curves for the confidence interval of 95% found no significant differences for the $K_{1/2}$ ($Dose_{first\_pulse}$=83.17ms [64.43ms, 101.9ms]; $Dose_{second\_pulse}$= 65.08ms [56.84ms, 73.32ms]), and for the n_{HILL} ($n_{HILL\_first\_pulse}$=2.039 [1.276, 2.802]; $n_{HILL\_second\_pulse}$=2.308

[1.686, 2.931]). But a difference was found for the EOG_{max} for the confidence interval of 95% ($p<0.05$) ($EOG_{max\_first\_pulse}$=1.01 [0.85, 1.16]; $EOG_{max\_second\_pulse}$=0.53 [0.48, 0.57]), which is a typical feature of desensitization. The goodness of the fit for the first and second EOG responses, respectively: SSE=175.4 and 27.37, R-square=0.9852 and 0.9925, Adjusted R-square=0.981 and 0.9903, and RMSE=5.006 and 1.977. In consequence of these results, it is possible that desensitization is involved in the reduction of the amplitude of the second response relative to the first response when increasing durations of pulses are used to generate a dose-response curve (Figure S1).

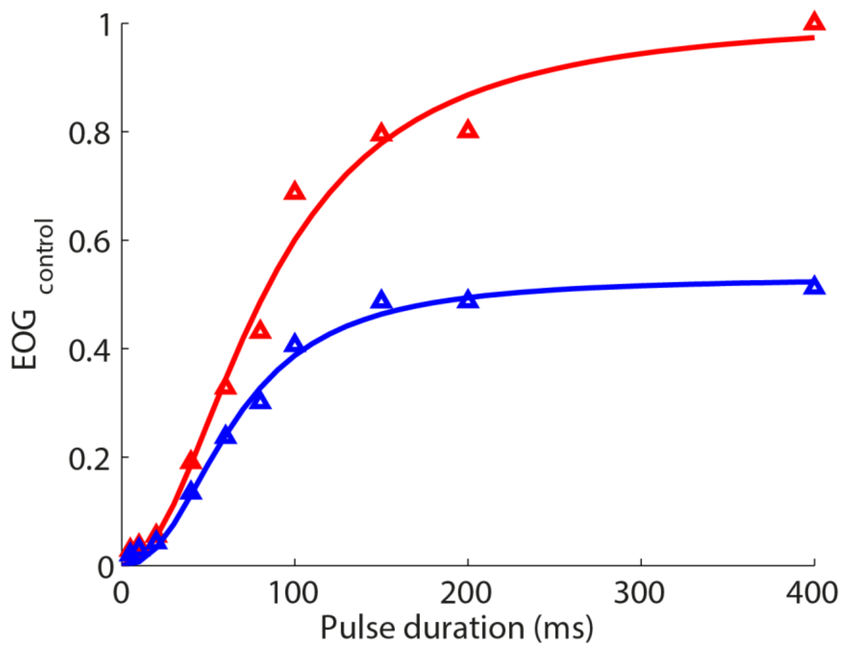

Figure S1:

Averaged EOG responses to the first pulse (red) and second pulse (blue) of odorant for different durations of stimulus. The dose-response curves were fitted with the Hill equation, and the parameters used to fit the non-adapted responses (red) and adapted responses (blue), respectively, are: $K_{1/2}$ =83.17ms and 65.08ms, n_{HILL}=2.039 and 2.308, and EOG_{max}=1.01 and 0.53.

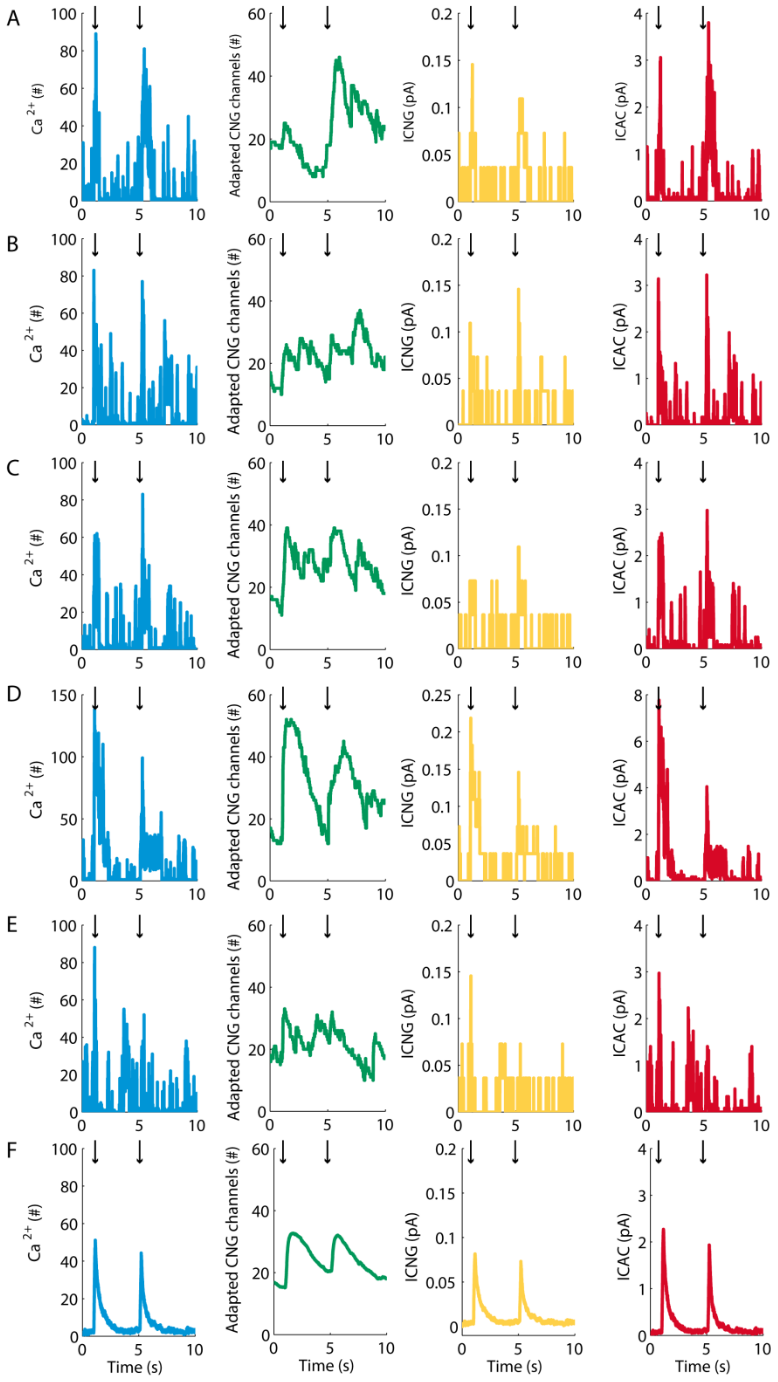

Figure S2. A-E: Examples of the time course of different components of the model obtained for single runs simulating the control situation. The arrows indicate the timing of the pulses of cAMP. F: Mean time course of the same components of the model calculated from 100 single runs.

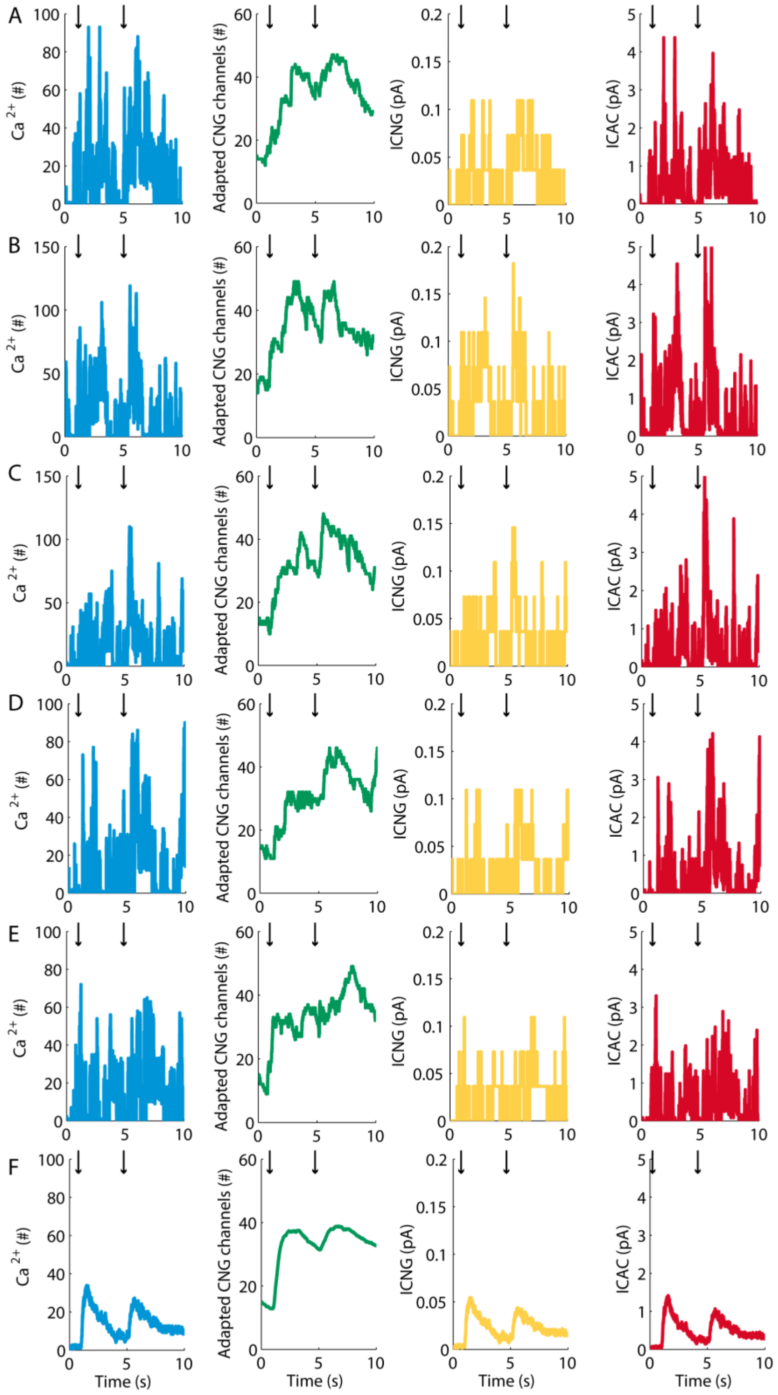

Figure S3. A-E: Effects of the simulated pharmacological treatment on the time course of different components of the model obtained for single runs. The arrows indicate the timing of the pulses of cAMP. F: Mean time course of the same components of the model calculated from 100 single runs.

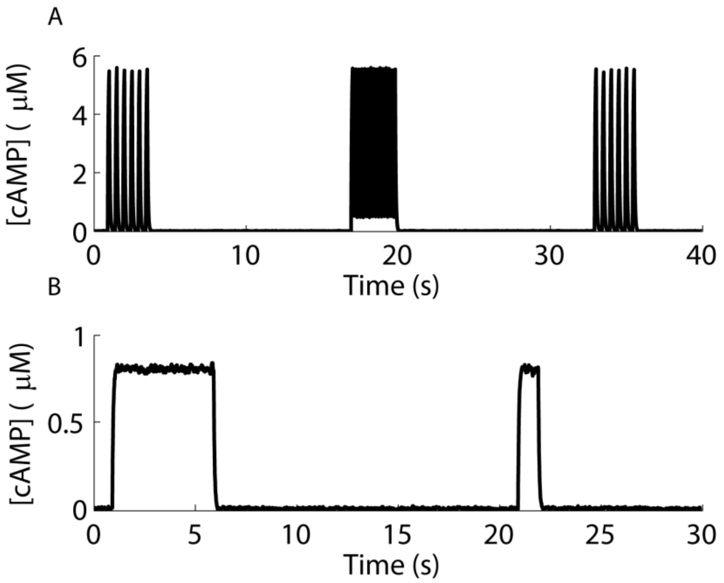

Figure S4:

Mean cAMP input used in the simulation of the EOG responses to pulses composed of slow (2 Hz) and fast (5 Hz) frequencies (A) and EOG responses to prolonged pulses of 5 s and 1 s (B). The result of the simulations is shown in Figure 5 D and E.

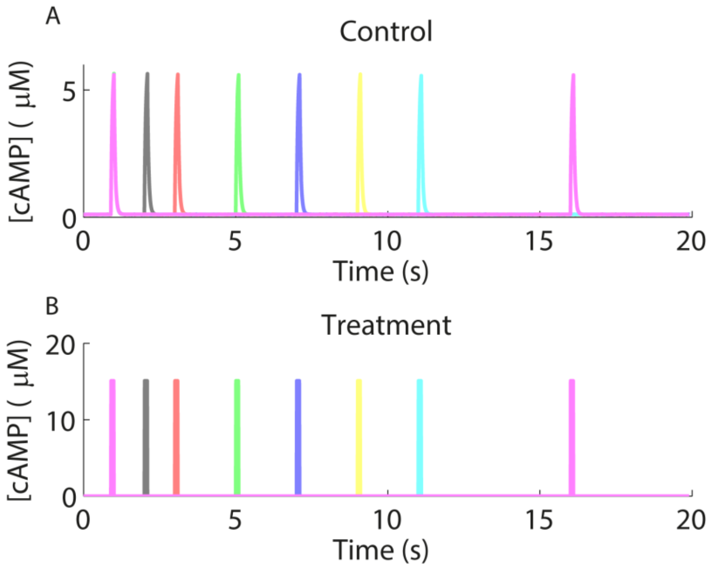

Figure S5:

Mean cAMP input used in the simulation of the EOG responses in the control (A) and treated condition (B) of Figure 6 C and D, respectively. The color code used is the same of Figure 6 C and D.

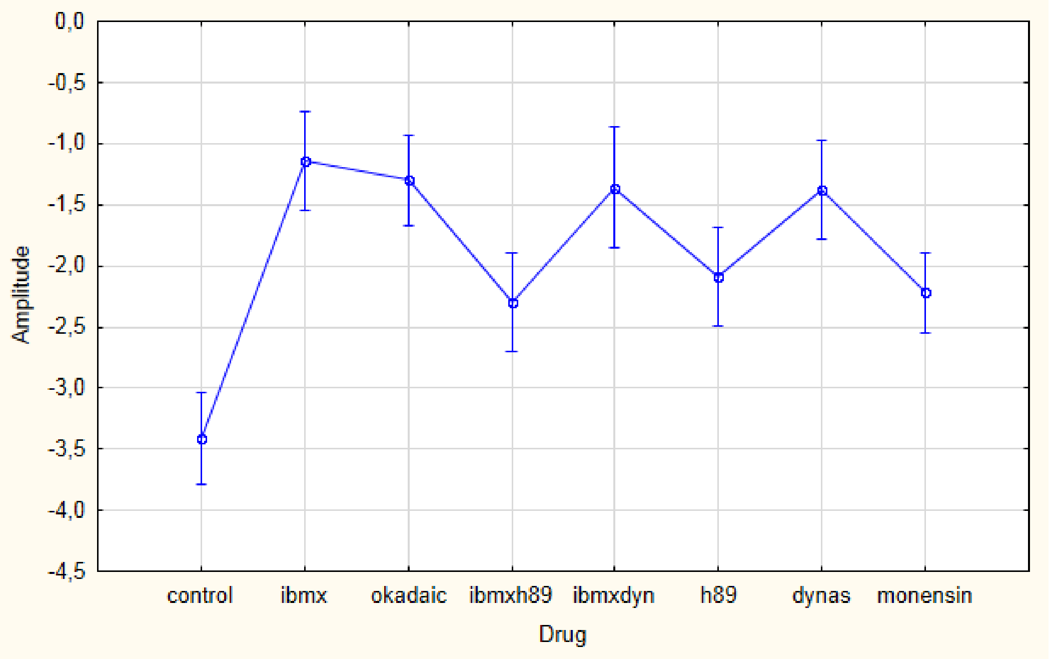

Figure S6:

Mean amplitude (mV) of the peak EOG responses to odorant puffs at different experimental conditions: control (n=49), ibmx (n=41), okadaic acid (n=47), ibmx+h89 (n=41), ibmx+dynasore (n=27), h89 (n=41), dynasore (n=41), and monensin (n=62). Vertical bars denote 0.95 confidence intervals.

Table S1

	**Latency**	**Rise Time**	**Decay Time**	**N**
cAMP	0.2007±0.0384	0.2800±0.078	0.7836±0.2381	8
cAMP wash	0.1807±0.0091	0.2570±0.0480	0.77409±0.2024	4
T-Test	N.S.	N.S.	N.S.	

The L, RT, and DT of the EOG responses to 100 ms puffs of odorants (mixture of acetophenone and cineole) were not significantly different in the treated (cAMP) and washed groups (cAMP wash) (T-Test, where $p<0.05$ was considered statistically significant). N.S. (Not Significant). Results are presented as mean±standard deviation.

List of abbreviations

OSN: olfactory sensory neurons

STA: short-term adaptation

DS: desensitization

EOG: electroolfactogram

PDEs: phosphodiesterases

PDE1C: phosphodiesterase 1C

PDE4A: phosphodiesterase 4A

cAMP: cyclic adenosine monophosphate

PKA: cAMP-dependent protein kinase

GPCR: G protein-coupled receptors

CNG channels: cyclic nucleotide gated channels

Ca^{2+}: calcium ion

Na^{+}: sodium ion

CAC channels: Ca^{2+}-activated chloride channels

Ca^{2+}/CaM: Ca^{2+}/Calmodulin

CaMKII: CaM Kinase II

NCKX: Na^{+}/ Ca^{2+} and K^{+} exchangers

NCX: K^{+}-independent Na^{+}/ Ca^{2+} exchangers

PMCA: plasma membrane Ca^{2+}-ATPases

GRK3: G-protein-coupled receptor kinase 3

ISI: inter-stimuli interval

PRA: percent of recovery of adaptation

ISI_{50}: half-maximum ISI

n: Hill coefficient

L: latency

RT: rise time

DT: decay time

M_{ISI50}: Mean ISI_{50}

M_L : Mean latency

M_{RT}: Mean rise time

M_{DT}: Mean decay time

ANOVA: analysis of variance

LSD: least significant difference

HSD: honestly significant difference

AC3: adenilate cyclases III

$G_{\alpha olf}$: alpha subunit of G_{olf} protein

DIP: dynamin inhibitory peptide

H89: N-[2-(p-Bromocinnamylamino)ethyl]-5-isoquinolinesulfonamide

IBMX: 3-isobutyl-1-methylxanthine

$IC_{50:}$ half maximal inhibitory concentration

DMSO: dimethyl sulfoxide

SEM: standard error of the mean

DARPP-32: dopamine-and cAMP-regulated neuronal phosphoprotein

PP1: protein phosphatase 1
